# Stage‐Dependent Prognostic Value of the Joint MASLD–NAFLD Fibrosis Score Phenotype Across the Cardiovascular–Kidney–Metabolic Syndrome Continuum: A Retrospective NHANES Cohort Analysis, 1999–2018

**DOI:** 10.1002/kjm2.70285

**Published:** 2026-08-26

**Authors:** Yi‐Fei Wang, Jian‐Li Zhang, Yun‐Fei Wang, Jun‐Peng Xiong, Rong‐Hui Yu, Ze‐Ping Hu

**Affiliations:** ^1^ Department of Cardiology, National Cardiovascular Disease Regional Center for Anhui The First Affiliated Hospital of Anhui Medical University Hefei Anhui China

**Keywords:** cardiovascular–kidney–metabolic syndrome, fatty liver index, metabolic dysfunction–associated steatotic liver disease, NAFLD fibrosis score, NHANES

## Abstract

The cardiovascular–kidney–metabolic (CKM) framework and metabolic dysfunction–associated steatotic liver disease (MASLD) describe a shared clinical continuum, but the prognostic value of joint fatty liver index (FLI)–NAFLD fibrosis score (NFS) phenotypes across CKM stages is uncertain. We analyzed 10 National Health and Nutrition Examination Survey cycles (1999–2018) linked to mortality through 2019. Among 31,317 adults, participants were classified as A (no MASLD/NFS‐low), B (MASLD/NFS‐low), C (no MASLD/NFS‐high), or D (MASLD/NFS‐high) and stratified by operational CKM Stages 0–4. Survey‐weighted survival analyses and Cox models incorporated 20‐imputation covariate analysis; exposure, outcome, follow‐up, and survey‐design fields were not imputed. During 291,392.5 person‐years, 4284 all‐cause and 1417 cardiovascular deaths occurred. All‐cause survival differed across phenotypes in Stages 2–4 (*p* < 0.001 for each), but not between A and B in Stage 1 (*p* = 0.695). At Stage 2, adjusted D‐versus‐A hazard ratios were 1.57 (95% CI 1.30–1.91) for all‐cause mortality and 2.09 (1.57–2.79) for cardiovascular mortality; corresponding Stage 4 estimates were 1.44 (1.21–1.70) and 1.25 (0.96–1.64). The full stage interaction was non‐estimable, and supported‐stage interaction *p* values were 0.113 and 0.122. Joint‐phenotype associations varied across supported CKM stages, but sparse early‐stage phenotypes, a small apparent discrimination gain, and nominal subgroup findings support external validation rather than immediate clinical deployment.

**Abbreviations**
AHA: American Heart Association
ALD: alcohol‐related liver disease
ALT: alanine aminotransferase
AST: aspartate aminotransferase
BMI: body mass index
CAP: controlled attenuation parameter
CI: confidence interval
CIF: cumulative incidence function
CKD: chronic kidney disease
CKM: cardiovascular–kidney–metabolic
CMRF: cardiometabolic risk factor
eGFR: estimated glomerular filtration rate
FIB‐4: fibrosis‐4 index
FLI: fatty liver index
HDL: high‐density lipoprotein
HR: hazard ratio
HSI: hepatic steatosis index
KM: Kaplan–Meier
LSM: liver stiffness measurement
MASLD: metabolic dysfunction–associated steatotic liver disease
MEC: mobile examination centre
MetALD: MASLD with increased alcohol intake
MI: multiple imputation
NCHS: National Center for Health Statistics
NDI: National Death Index
NFS: NAFLD fibrosis score
NHANES: National Health and Nutrition Examination Survey
PSU: primary sampling unit
VCTE: vibration‐controlled transient elastography

## 1 Introduction

The cardiovascular–kidney–metabolic (CKM) syndrome and metabolic dysfunction–associated steatotic liver disease (MASLD) describe intersecting manifestations of systemic metabolic dysfunction. The American Heart Association framework operationalizes CKM health from absence of recognized risk factors through adiposity, metabolic and kidney abnormalities, and clinical cardiovascular disease [1, 2]. In parallel, the multisociety MASLD nomenclature defines steatotic liver disease affirmatively in the presence of cardiometabolic risk [3, 4]. Cardiovascular events and mortality are major outcomes among people with steatotic liver disease, and increasing fibrosis burden is consistently associated with adverse prognosis in observational cohorts [5, 6, 7, 8, 9, 10, 11, 12]. FLI and NFS offer scalable non‐invasive proxies for probable steatosis and fibrosis risk, respectively, but neither is a diagnostic substitute for imaging or histology [13, 14, 15, 16].

Despite this conceptual convergence, the prognostic contribution of joint liver‐proxy phenotyping across the CKM continuum remains uncertain. Previous studies have generally examined steatosis or fibrosis scores as stand‐alone exposures, used cohorts with restricted clinical spectra, or reported overall associations without resolving sparse stage‐by‐phenotype cells [7, 8, 9, 10, 11]. Interpretation is particularly difficult because NFS incorporates age, adiposity, glycemic status, aminotransferases, platelets, and albumin, while operational CKM staging also incorporates adiposity, metabolic disease, kidney dysfunction, and cardiovascular disease. These shared constructs can alter comparison groups and complicate claims of independent or stage‐specific biological effects [17, 18]. Competing death, proxy misclassification, missing data, treatment differences, and sparse early‐stage outcomes add further uncertainty.

To address these gaps, we rebuilt 10 NHANES cycles from the original component files and linked the resulting cohort to public‐use mortality follow‐up. Our objectives were: (i) to document a reproducible participant flow and the correct 20‐year pooled‐weight construction; (ii) to describe joint phenotype prevalence across operational CKM stages and survey cycles; (iii) to distinguish unadjusted all‐cause survival and cardiovascular cumulative incidence from adjusted cause‐specific mortality associations; (iv) to evaluate supported stage‐specific phenotype contrasts and continuous NFS dose–response; (v) to examine cardiometabolic risk‐factor burden and exploratory D‐versus‐A subgroup heterogeneity; and (vi) to assess apparent incremental prediction, alternative steatosis/fibrosis proxies, alcohol‐etiology classification, and 2017–2018 vibration‐controlled transient elastography (VCTE) sensitivity analyses.

## 2 Methods

### 2.1 Study Design and Population

We conducted a retrospective cohort analysis of prospectively collected examination data from 10 consecutive NHANES cycles, from 1999–2000 through 2017–2018. NHANES uses a complex, multistage, stratified probability design to represent the civilian, non‐institutionalized U.S. population. Eligible participants were linked to the 2019 public‐use National Death Index (NDI) mortality file, with follow‐up through December 31, 2019 [19, 20, 21].

**FIGURE 1:**
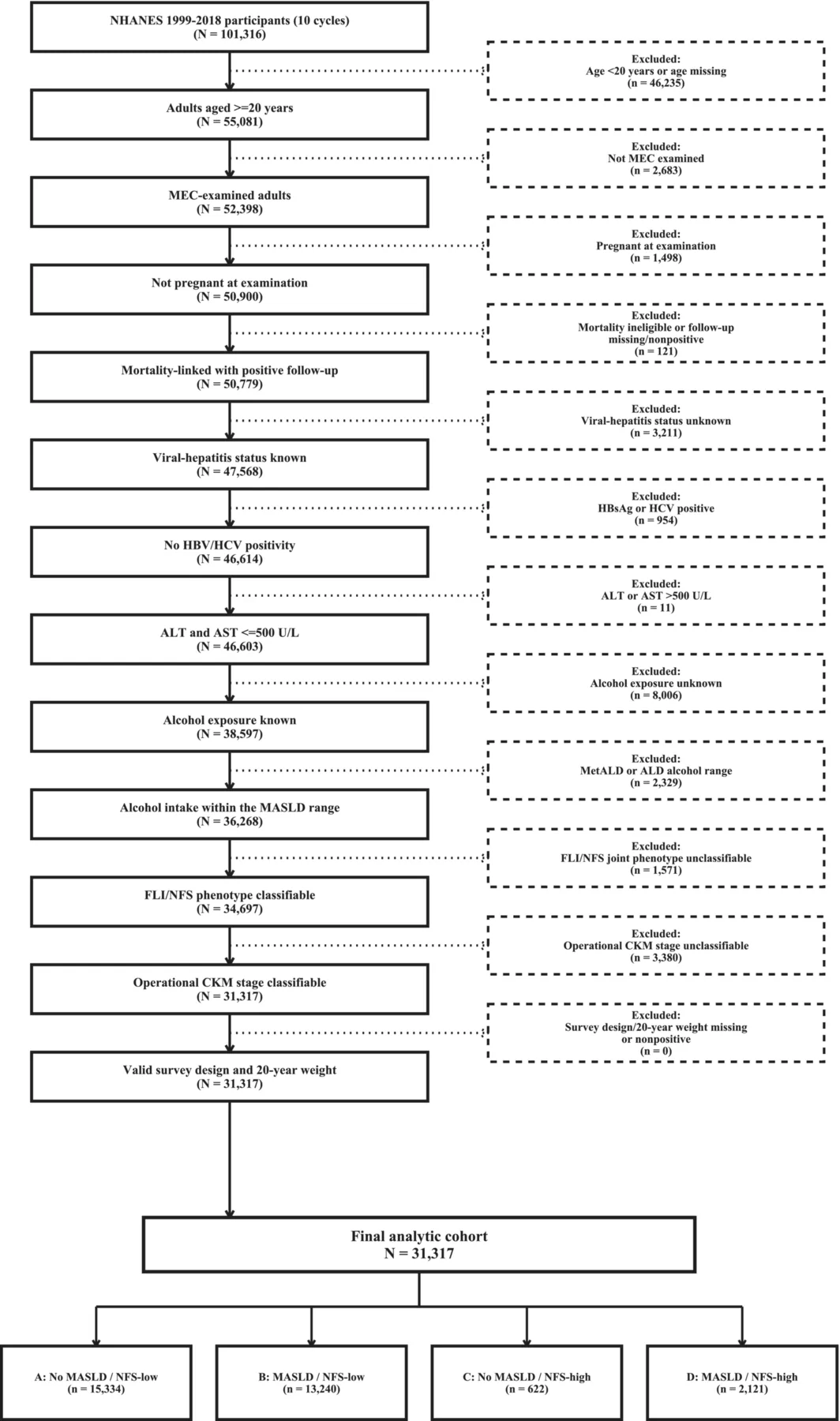
Participant‐selection flowchart.

From 101,316 source participants, we sequentially excluded age < 20 years or missing age (*n* = 46,235), no mobile examination centre examination (*n* = 2683), pregnancy (*n* = 1498), mortality ineligibility or non‐positive/missing follow‐up (*n* = 121), unknown viral‐hepatitis status (*n* = 3211), positive hepatitis B surface antigen or hepatitis C virus status (*n* = 954), alanine or aspartate aminotransferase > 500 U/L (*n* = 11), unknown alcohol exposure (*n* = 8006), MetALD/ALD‐range intake (*n* = 2329), an unclassifiable FLI/NFS phenotype (*n* = 1571), or an unclassifiable operational CKM stage (*n* = 3380). No final participant was excluded for missing or non‐positive survey‐design data. The final cohort comprised 31,317 adults. Each source participant was assigned one mutually exclusive first‐exclusion reason (Figure 1; Table S1).

The MEC examination weight was used because the primary FLI incorporated triglycerides from the standard biochemistry component. The 1999–2002 4‐year examination weight was scaled as WTMEC4YR × 4/20; each 2003–2018 2‐year examination weight was scaled as WTMEC2YR × 2/20 [19]. Masked strata and primary sampling units were nested within survey cycle. The fasting‐FLI sensitivity analysis used the corresponding pooled fasting‐subsample weight (Table S2).

### 2.2 Assessment of Exposure

FLI was calculated as 100 × exp(*z*)/(1 + exp(*z*)), where *z* = 0.953 × ln(triglycerides [mg/dL]) + 0.139 × body mass index + 0.718 × ln(gamma‐glutamyl transferase [U/L]) + 0.053 × waist circumference [cm] − 15.745; FLI ≥ 60 indicated probable steatosis [13]. MASLD required FLI‐defined steatosis plus at least one of five cardiometabolic criteria, within the MASLD alcohol range [3, 22, 23]. NFS was calculated as −1.675 + 0.037 × age + 0.094 × body mass index + 1.13 × operational glycaemia indicator + 0.99 × AST/ALT − 0.013 × platelet count − 0.66 × albumin; NFS > 0.676 defined NFS‐high [14]. For cross‐cycle harmonization, the glycaemia term denoted self‐reported or treated diabetes or glycated hemoglobin ≥ 5.7%, because fasting glucose was not consistently available.

Participants were assigned to four mutually exclusive phenotypes: A, no MASLD/NFS‐low; B, MASLD/NFS‐low; C, no MASLD/NFS‐high; and D, MASLD/NFS‐high. NFS‐low was not interpreted as histological absence of fibrosis. Average ethanol intake was calculated from drinking frequency, drinks per drinking day, and 14 g ethanol per standard drink. MASLD‐range intake was ≤ 20 g/day in women and ≤ 30 g/day in men; MetALD‐range intake was > 20–50 and > 30–60 g/day, respectively; higher intake was ALD‐range. MetALD/ALD and unknown alcohol exposure were excluded before the primary phenotype was defined and were not placed in the no‐MASLD reference group.

Operational CKM stage was assigned from the highest stage downward. Stage 4 denoted self‐reported clinical cardiovascular disease; Stage 3 denoted high‐risk kidney disease (estimated glomerular filtration rate < 45 mL/min/1.73 m^2^ or urine albumin‐to‐creatinine ratio ≥ 300 mg/g) without clinical cardiovascular disease; Stage 2 denoted metabolic risk or moderate kidney disease; Stage 1 denoted adiposity alone (body mass index ≥ 25 kg/m^2^ or waist circumference ≥ 94 cm in men or ≥ 80 cm in women); and Stage 0 denoted none of these. Estimated glomerular filtration rate used the 2021 race‐free CKD‐EPI creatinine equation [24]. This operational implementation did not reproduce every subclinical‐cardiovascular element of the full AHA Stage 3 definition [1]. Cardiometabolic risk‐factor (CMRF) count summed adiposity, dysglycemia, elevated blood pressure, elevated triglycerides, and low high‐density‐lipoprotein cholesterol or related treatment (range 0–5).

### 2.3 Ascertainment of Outcomes

The primary outcome was all‐cause mortality. Cardiovascular mortality was identified from the public‐use UCOD_LEADING categories 001 (diseases of heart) and 005 (cerebrovascular disease); other deaths were censored in cause‐specific Cox models [20, 21]. Follow‐up extended from MEC examination to death or administrative censoring. The public‐use mortality file does not contain the detailed underlying or multiple‐cause ICD‐10 codes required to identify liver‐related death; the residual other‐cause category was therefore not relabeled as liver mortality.

### 2.4 Covariates

The prespecified adjustment set comprised age, sex, race or ethnicity, educational attainment, family income‐to‐poverty ratio, and smoking status. These variables were selected as harmonizable demographic, socioeconomic, and behavioral confounders. Biomarkers and diagnoses already used to construct FLI, NFS, MASLD, CMRF burden, or CKM stage were not re‐entered into the primary model, thereby avoiding duplicate adjustment for exposure and stratification components. Medication subgroup variables were retained with their source labels, including lipid‐lowering medication use.

### 2.5 Statistical Analysis

Analyses were prespecified and proceeded in the following sequence.

#### 2.5.1 Descriptive Statistics

Continuous variables are reported as survey‐weighted means and categorical variables as survey‐weighted percentages; sample sizes and event counts are unweighted. Phenotype prevalence was estimated by operational CKM stage and by each of the 10 survey cycles, with design‐based 95% confidence intervals.

#### 2.5.2 Missing Data

Twenty chained‐equation imputations (10 iterations) were created for education, income‐to‐poverty ratio, marital status, and smoking; marital status served as an imputation auxiliary variable and was not included in the primary adjustment set. Phenotype, MASLD, NFS, CKM stage, outcomes, follow‐up, weights, strata, primary sampling units, and other analysis‐defining fields were protected from imputation and verified as identical across all completed data sets. Each completed data set received a cycle‐aware survey design, and coefficients and covariance matrices were combined using Rubin's rules.

#### 2.5.3 Survival Analysis

Unadjusted all‐cause survival and cardiovascular cumulative incidence were computed once from the protected analysis fields rather than repeatedly across imputed data sets. All‐cause survival was estimated using survey::svykm with the 20‐year MEC weights and cycle‐nested strata and primary sampling units. Supported curves comprised A/B in Stage 1 and A–D in Stages 2–4; Stage 0 was structurally unsupported. Curves required at least 30 participants and five deaths, displayed unweighted numbers at risk at 0, 5, 10, and 15 years, and were truncated when fewer than 10 participants remained at risk. Design‐based survey::svylogrank tests were reported. Cardiovascular cumulative incidence was estimated descriptively with 20‐year MEC‐weighted Aalen–Johansen estimates, treating non‐cardiovascular death as a competing event. No Gray test, Fine–Gray model, subdistribution hazard ratio, inferential cumulative‐incidence *p* value, or design‐based cumulative‐incidence confidence interval was claimed.

#### 2.5.4 Regression and Estimability

Stage‐specific survey‐weighted Cox models estimated all‐cause and cause‐specific cardiovascular hazard ratios. In Stages 2–4, B, C, and D versus A coefficients came from the same four‐level phenotype model. Stage 1 B versus A was evaluated in a dedicated NFS‐low A/B model; Stage 0 and sparse contrasts were not estimated. Each comparison group required at least 30 participants and five outcome events. The global phenotype‐by‐stage interaction was attempted across Stages 0–4; because early stages lacked all four phenotypes, a separate interaction restricted to the common Stages 2–4 support set was reported without representing it as a replacement for the unsupported full interaction. Direct B‐versus‐A and D‐versus‐C models were fitted separately within NFS strata.

#### 2.5.5 Restricted Cubic Spline Analysis

Continuous NFS was represented by a natural/restricted cubic spline with stage‐specific knots at the 5th, 35th, 65th, and 95th percentiles. Hazard ratios were referenced to the within‐stage median NFS, and the NFS = 0.676 threshold was marked for interpretation. Only Stages 2–4 met the prespecified NFS‐low/high support rule; entire Stages 0–1 panels were suppressed rather than extrapolated.

#### 2.5.6 Incremental Predictive Value

Base, +MASLD, +NFS, and +joint Cox models used mean‐one‐rescaled survey weights and cluster‐robust standard errors. Apparent weighted Harrell *C* and pseudo‐likelihood diagnostics were averaged across imputations. These were in‐sample exploratory measures; no externally validated discrimination, calibration, time‐dependent area under the curve, integrated discrimination improvement, net reclassification improvement, Brier score, or decision‐curve net benefit was estimated [25, 26].

#### 2.5.7 Subgroup Analyses

D versus A was estimated within the combined CKM Stages 2–4 support set across 11 prespecified dimensions: age, sex, race or ethnicity, diabetes, body mass index, hypertension, chronic kidney disease, smoking, lipid‐lowering medication, antihypertensive medication, and glucose‐lowering medication. Models included categorical CKM stage and the primary adjustment set with the stratifying variable removed where applicable. Medication strata were observed‐status available‐case analyses. Heavy alcohol was not fitted because MetALD/ALD and unknown alcohol exposure had been excluded before primary cohort definition. Interaction *p* values were nominal and not adjusted for multiplicity.

#### 2.5.8 Sensitivity Analyses

Sensitivity analyses included complete‐case and 2‐year landmark survey Cox models, hepatic steatosis index and fasting‐FLI alternatives, FIB‐4, CMRF burden, HR‐as‐risk‐ratio E‐values [27], alcohol‐etiology audits, and analysis execution and warning ledgers. In 2017–2018, reliable VCTE required at least 10 valid stiffness measurements, liver‐stiffness IQR/median < 30%, and observed controlled attenuation parameter (CAP) and liver‐stiffness measurement [28]. FLI ≥ 60 was compared with CAP ≥ 248 dB/m, and NFS > 0.676 with liver‐stiffness thresholds ≥ 8 and ≥ 12 kPa [22, 23, 29]. Weighted diagnostic proportions and unweighted DeLong areas under the curve were treated as cross‐sectional proxy comparisons rather than mortality‐prediction validation.

#### 2.5.9 Software

Analyses were conducted in R 4.4.1 using survey, mice, survival, splines, and related packages. Two‐sided *p* < 0.05 denoted nominal statistical significance. No multiplicity correction was applied to exploratory subgroup interaction tests.

## 3 Results

### 3.1 Baseline Characteristics

The final cohort included 31,317 adults, with 4284 all‐cause deaths and 1417 cardiovascular deaths during 291,392.5 person‐years. Median follow‐up was 8.67 years (interquartile range 4.58–13.42), weighted mean age was 47.9 years, and 52.9% were women. Operational CKM stage counts were 1768, 2806, 22,562, 820, and 3361 for Stages 0–4, respectively (Figure 1, Table 1). Weighted mean age rose monotonically from 35.9 years in Stage 0 to 64.6 years in Stage 4. Body mass index, glycated hemoglobin, and CMRF count were higher in later stages but peaked in Stage 3 (30.5 kg/m^2^, 6.49%, and 3.57, respectively) before declining modestly in Stage 4 (30.1 kg/m^2^, 6.02%, and 3.49).

**TABLE 1:** Survey‐weighted characteristics of U.S. adults by operational CKM stage, NHANES 1999–2018 (*N* = 31,317).

Characteristic	CKM Stage 0	CKM Stage 1	CKM Stage 2	CKM Stage 3	CKM Stage 4
Unweighted no.	1768	2806	22,562	820	3361
Age, years	35.9	40.0	48.1	62.5	64.6
Women, %	51.8	64.1	51.5	61.4	48.4
Body mass index, kg/m^2^	21.6	28.0	29.9	30.5	30.1
Systolic blood pressure, mmHg	110.4	112.0	125.1	138.3	131.0
HbA1c, %	5.14	5.19	5.65	6.49	6.02
Triglycerides, mg/dL	74.8	83.9	171.6	195.4	167.1
HDL cholesterol, mg/dL	63.7	61.6	49.5	51.6	50.4
eGFR, mL/min/1.73 m^2^	105.8	102.6	96.6	59.5	77.5
Fatty liver index	9.0	37.4	59.7	63.0	63.9
NAFLD fibrosis score	−3.15	−2.51	−1.83	−0.50	−0.47
CMRF count	0.00	1.00	2.88	3.57	3.49
Alcohol, g/day	5.3	4.8	4.1	2.4	3.3

*Note:* Values are survey‐weighted means or percentages unless stated otherwise; the no. row contains unweighted counts. Estimates used the audited 20‐year MEC weights. CMRF count was available for 27,027 participants and was not imputed.

Abbreviations: CKM, cardiovascular–kidney–metabolic; CMRF, cardiometabolic risk factor; eGFR, estimated glomerular filtration rate; FLI, fatty liver index; HbA1c, glycated hemoglobin; HDL, high‐density lipoprotein; MEC, mobile examination centre; NFS, NAFLD fibrosis score; NHANES, National Health and Nutrition Examination Survey.

Phenotype counts were 15,334 (A), 13,240 (B), 622 (C), and 2121 (D). When stratified by phenotype, Group C was the oldest (weighted mean age 75.4 years) and had the lowest mean estimated glomerular filtration rate (67.6 mL/min/1.73 m^2^), whereas Group D had the highest body mass index (38.2 kg/m^2^), glycated hemoglobin (6.50%), FLI (90.0), NFS (1.42), and CMRF count (4.02). These patterns emphasize that Group C and Group D captured clinically different routes to NFS‐high status (Table 2; Tables S4 and S5).

**TABLE 2:** Survey‐weighted joint MASLD–NFS phenotype prevalence by operational CKM stage, NHANES 1999–2018 (*N* = 31,317).

CKM stage	Phenotype	Unweighted no.	Weighted prevalence, % (95% CI)
Stage 0	A	1767	100.0 (99.9–100.0)
Stage 0	B	0	0.0 (0.0–0.0)
Stage 0	C	1	0.0 (0.0–0.1)
Stage 0	D	0	0.0 (0.0–0.0)
Stage 1	A	2163	77.9 (76.0–79.9)
Stage 1	B	628	21.7 (19.8–23.6)
Stage 1	C	3	0.1 (0.0–0.1)
Stage 1	D	12	0.3 (0.1–0.5)
Stage 2	A	9981	44.6 (43.6–45.6)
Stage 2	B	10,963	50.3 (49.3–51.3)
Stage 2	C	348	0.9 (0.8–1.0)
Stage 2	D	1270	4.2 (3.9–4.6)
Stage 3	A	267	34.3 (29.9–38.8)
Stage 3	B	327	40.9 (37.0–44.8)
Stage 3	C	51	6.2 (4.0–8.3)
Stage 3	D	175	18.6 (15.0–22.2)
Stage 4	A	1156	34.7 (32.5–36.9)
Stage 4	B	1322	41.5 (39.1–43.8)
Stage 4	C	219	5.5 (4.5–6.4)
Stage 4	D	664	18.4 (16.8–20.0)

*Note:* Phenotypes were defined by the joint distribution of MASLD and NFS category: A, no MASLD/NFS‐low; B, MASLD/NFS‐low; C, no MASLD/NFS‐high; and D, MASLD/NFS‐high. NFS‐high was defined as > 0.676. Counts are unweighted and percentages are survey weighted.

Abbreviations: CI, confidence interval; CKM, cardiovascular–kidney–metabolic; MASLD, metabolic dysfunction‐associated steatotic liver disease; NFS, NAFLD fibrosis score; NHANES, National Health and Nutrition Examination Survey.

### 3.2 Epidemiological Burden of MASLD‐NFS Phenotypes Across the CKM Continuum

**FIGURE 2:**
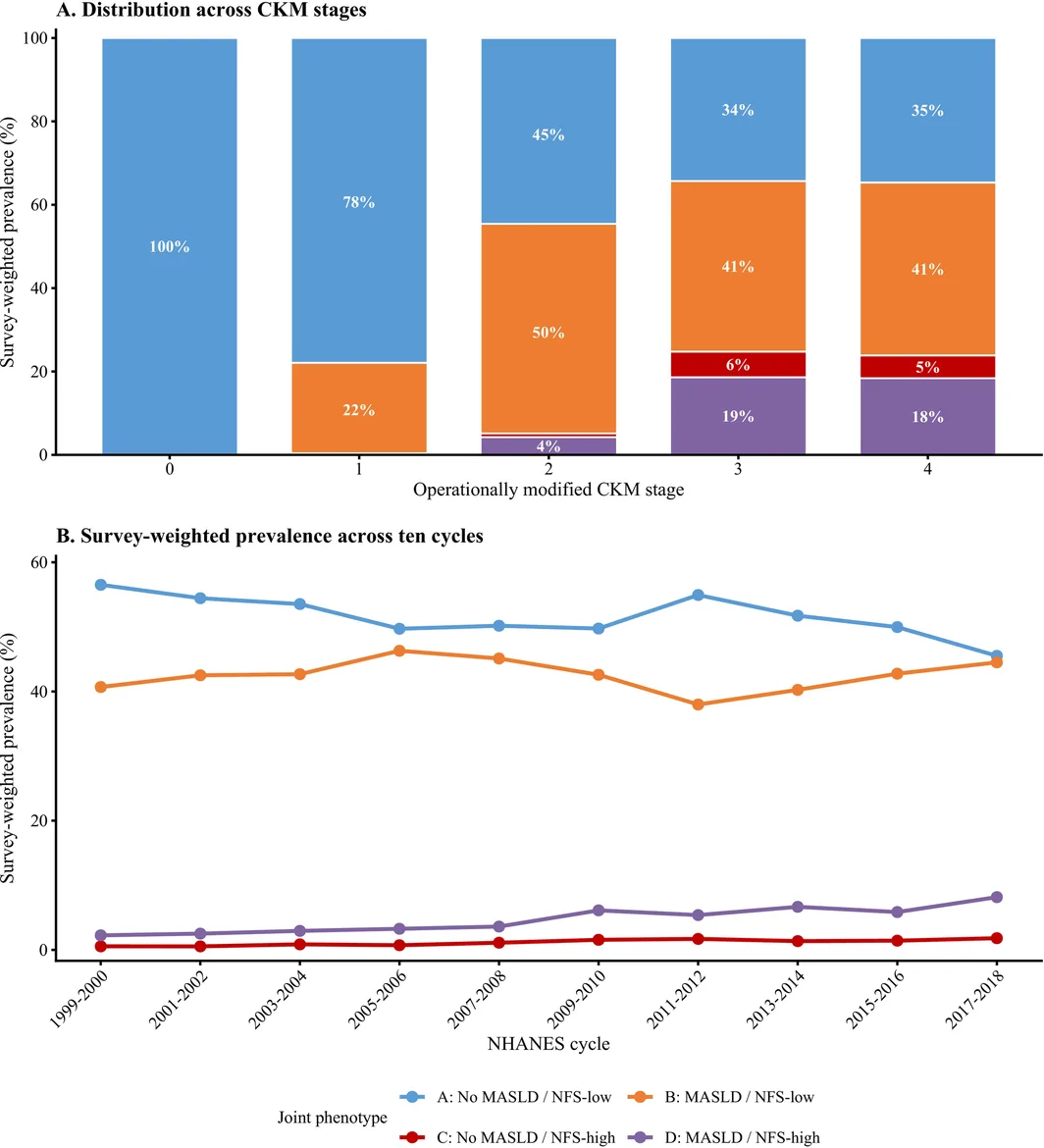
Survey‐weighted joint‐phenotype distribution and temporal pattern. Panel A shows the four joint MASLD–NFS phenotypes across operational CKM stages; panel B shows their survey‐weighted prevalence across the 10 NHANES cycles.

Phenotype prevalence shifted substantially across operational CKM stages (Figure 2A, Table 2). Phenotype A accounted for 100.0% of weighted Stage 0 and 77.9% of Stage 1, but 44.6%, 34.3%, and 34.7% of Stages 2, 3, and 4, respectively. Conversely, phenotype D increased from 4.2% (95% CI 3.9–4.6) in Stage 2 to 18.6% (15.0–22.2) in Stage 3 and 18.4% (16.8–20.0) in Stage 4. Phenotype C remained uncommon, ranging from 0.9% in Stage 2 to 6.2% in Stage 3 and 5.5% in Stage 4.

Across the 10 survey cycles, weighted phenotype D prevalence increased from 2.3% (95% CI 1.6–2.9) in 1999–2000 to 8.2% (7.1–9.2) in 2017–2018, while phenotype A declined from 56.5% to 45.5% (Figure 2B; Table S3; Figure S1). These cycle‐specific estimates describe changing population composition and were not interpreted as individual‐level progression.

### 3.3 Survival and Cumulative Incidence by CKM Stage

**FIGURE 3:**
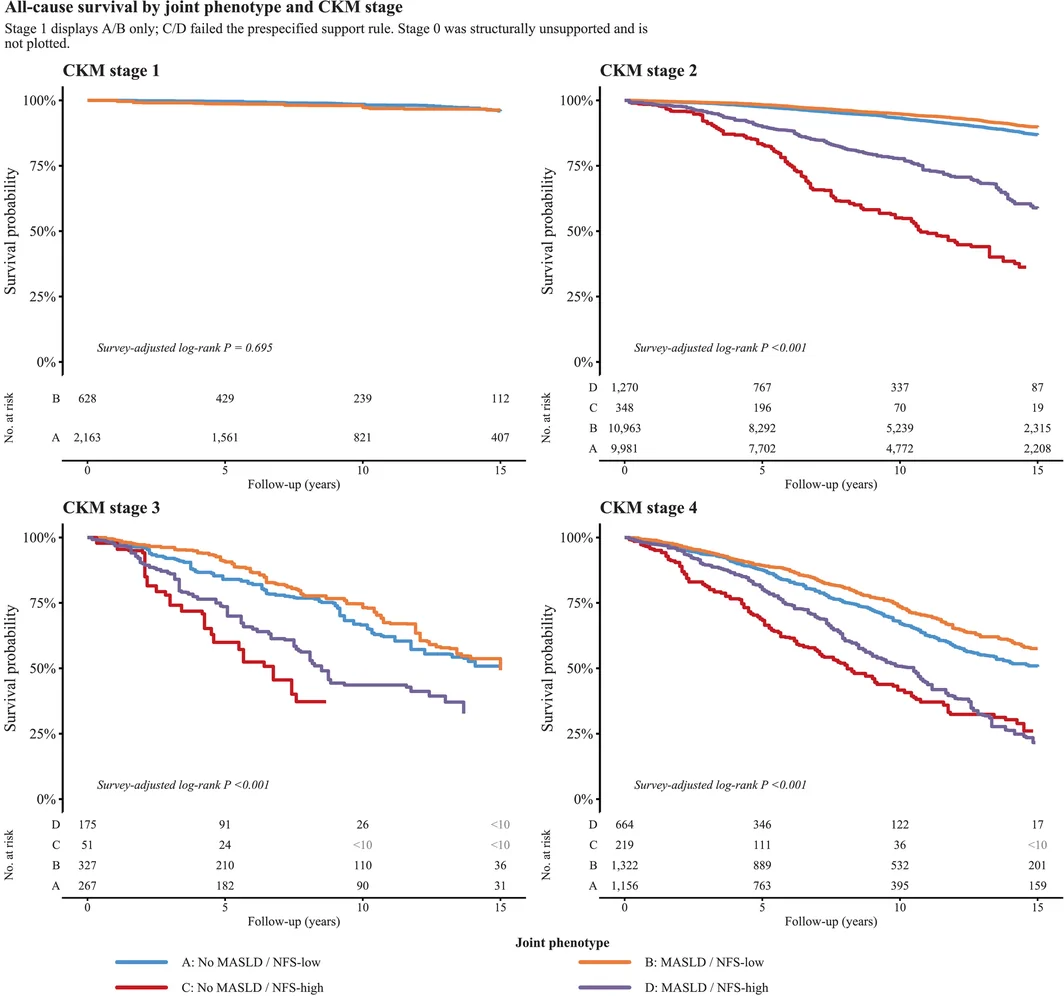
Unadjusted survey‐weighted Kaplan–Meier estimates of all‐cause survival by joint phenotype and operational CKM stage.

Survey‐weighted Kaplan–Meier curves showed little separation between A and B in Stage 1 (design‐based log‐rank *p* = 0.695) but clear overall phenotype differences in Stages 2, 3, and 4 (*p* < 0.001 for each; Figure 3). At 10 years, survival ranged from 97.3% to 98.5% across the two Stage 1 curves and from 55.1% to 94.8% across the four Stage 2 curves. In Stage 3, one of four phenotype curves had fewer than 10 participants still at risk by 10 years; among the remaining three, survival ranged from 43.6% to 73.2%. Stage 4 survival ranged from 41.7% to 73.4% across the four curves.

**FIGURE 4:**
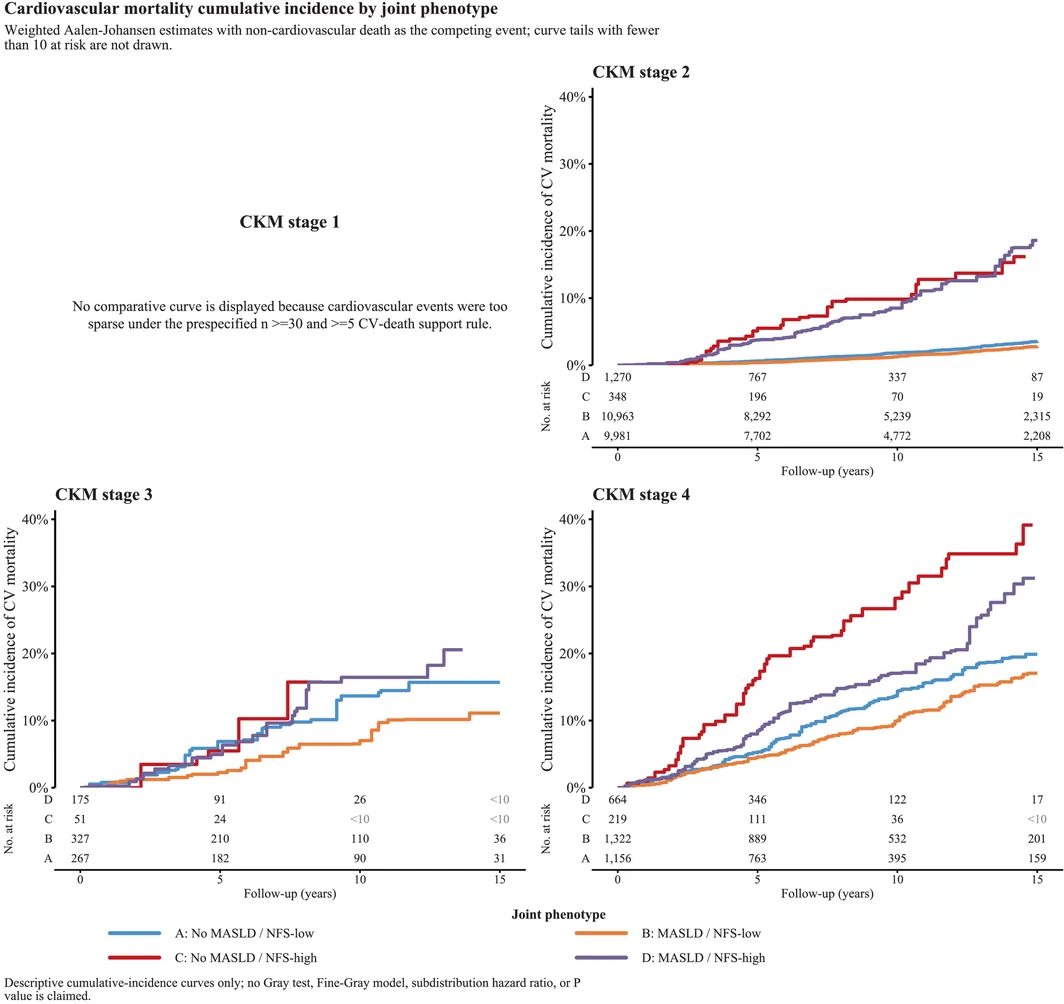
Descriptive 20‐year MEC‐weighted Aalen–Johansen cumulative incidence of cardiovascular death, treating non‐cardiovascular death as a competing event.

The descriptive weighted Aalen–Johansen curves likewise showed increasing absolute cardiovascular mortality with follow‐up in supported Stages 2–4 (Figure 4). At 10 years, cumulative incidence ranged from 1.3% to 9.9% across the four Stage 2 curves; in Stage 3, among the three curves with at least 10 participants still at risk, it ranged from 7.0% to 16.4%; and in Stage 4 it ranged from 10.0% to 28.2% across four curves. Stage 1 was retained as explicitly unsupported. These estimates incorporated competing non‐cardiovascular death but did not provide a Gray test, Fine–Gray model, or design‐based cumulative‐incidence confidence interval.

### 3.4 Association of Joint Phenotype With Mortality From Cox Regression

Adjusted all‐cause associations differed in magnitude across supported stage‐specific contrasts (Table 3; Figures S7 and S8). In Stage 1, B versus A was not associated with mortality (HR 0.99, 95% CI 0.49–1.99; *p* = 0.978). In Stage 2, B, C, and D versus A yielded HRs of 0.95 (0.86–1.06), 1.44 (1.18–1.77), and 1.57 (1.30–1.91), respectively. The corresponding estimates in Stage 3 were 1.00 (0.74–1.34), 1.74 (0.95–3.21), and 1.87 (1.31–2.68), and in Stage 4 were 1.15 (0.96–1.38), 1.27 (0.97–1.65), and 1.44 (1.21–1.70). Stage 0 and sparse Stage 1 C/D contrasts were not estimated.

**TABLE 3:** Adjusted hazard ratios for all‐cause mortality associated with joint MASLD–NFS phenotype within operational CKM stages, NHANES 1999–2018.

Contrast	No.	Deaths	Adjusted HR (95% CI)	*p*	Reason if not estimable
CKM Stage 0					
Model not estimable	1768	50	Not estimable	—	Stage 0 phenotype support is A = 1767; B = 0; C = 1; D = 0; the four‐level phenotype model is structurally non‐estimable
CKM Stage 1					
B vs. A	2791	74	0.99 (0.49–1.99)	0.978	—
C vs. A	2806	80	Not estimable	—	Sparse contrast cell(s); minimum required per group: *n* ≥ 30 and events ≥ 5. Observed A *n* = 2163/events = 55; C *n* = 3/events = 2
D vs. A	2806	80	Not estimable	—	Sparse contrast cell(s); minimum required per group: *n* ≥ 30 and events ≥ 5. Observed A *n* = 2163/events = 55; D *n* = 12/events = 4
CKM Stage 2					
B vs. A	22,562	2541	0.95 (0.86–1.06)	0.390	—
C vs. A	22,562	2541	1.44 (1.18–1.77)	< 0.001	—
D vs. A	22,562	2541	1.57 (1.30–1.91)	< 0.001	—
CKM Stage 3					
B vs. A	820	304	1.00 (0.74–1.34)	0.984	—
C vs. A	820	304	1.74 (0.95–3.21)	0.075	—
D vs. A	820	304	1.87 (1.31–2.68)	< 0.001	—
CKM Stage 4					
B vs. A	3361	1309	1.15 (0.96–1.38)	0.126	—
C vs. A	3361	1309	1.27 (0.97–1.65)	0.077	—
D vs. A	3361	1309	1.44 (1.21–1.70)	< 0.001	—

*Note:* Survey‐weighted Cox models were combined across 20 covariate‐only imputations using Rubin's rules and adjusted for age, sex, race or ethnicity, educational attainment, family income‐to‐poverty ratio, and smoking. In Stages 2–4, B/C/D versus A estimates came from one four‐level phenotype model. Stage 1 B versus A came from a dedicated NFS‐low model; sparse C/D contrasts were not estimated. Stage 0 was structurally non‐estimable. The corresponding forest plot is Figure S2.

Abbreviations: CI, confidence interval; CKM, cardiovascular–kidney–metabolic; HR, hazard ratio; MASLD, metabolic dysfunction‐associated steatotic liver disease; MI, multiple imputation; NFS, NAFLD fibrosis score; NHANES, National Health and Nutrition Examination Survey.

For cause‐specific cardiovascular mortality, Stage 2 D versus A was associated with higher risk (HR 2.09, 95% CI 1.57–2.79; *p* < 0.001), whereas B versus A (0.98, 0.81–1.20) and C versus A (1.28, 0.85–1.93) were not (Table 4; Figure S9). D‐versus‐A estimates were 1.55 (0.87–2.76; *p* = 0.136) in Stage 3 and 1.25 (0.96–1.64; *p* = 0.099) in Stage 4. The complete Stages 0–4 interaction was structurally non‐estimable; within the common Stages 2–4 support set, phenotype‐by‐stage interaction *p* values were 0.113 for all‐cause and 0.122 for cardiovascular mortality.

**TABLE 4:** Adjusted cause‐specific hazard ratios for cardiovascular mortality associated with joint MASLD–NFS phenotype within operational CKM stages, NHANES 1999–2018.

Contrast	No.	Deaths	Adjusted HR (95% CI)	*p*	Reason if not estimable
CKM Stage 0					
Model not estimable	1768	10	Not estimable	—	Stage 0 phenotype support is A = 1767; B = 0; C = 1; D = 0; the four‐level phenotype model is structurally non‐estimable.
CKM Stage 1					
B vs. A	2791	15	Not estimable	—	Sparse contrast cell(s); minimum required per group: *n* ≥ 30 and events ≥ 5. Observed A *n* = 2163/events = 12; B *n* = 628/events = 3
C vs. A	2806	17	Not estimable	—	Sparse contrast cell(s); minimum required per group: *n* ≥ 30 and events ≥ 5. Observed A *n* = 2163/events = 12; C *n* = 3/events = 1
D vs. A	2806	17	Not estimable	—	Sparse contrast cell(s); minimum required per group: *n* ≥ 30 and events ≥ 5. Observed A *n* = 2163/events = 12; D *n* = 12/events = 1
CKM Stage 2					
B vs. A	22,562	748	0.98 (0.81–1.20)	0.878	—
C vs. A	22,562	748	1.28 (0.85–1.93)	0.235	—
D vs. A	22,562	748	2.09 (1.57–2.79)	< 0.001	—
CKM Stage 3					
B vs. A	820	90	0.74 (0.40–1.35)	0.326	—
C vs. A	820	90	1.06 (0.39–2.90)	0.906	—
D vs. A	820	90	1.55 (0.87–2.76)	0.136	—
CKM Stage 4					
B vs. A	3361	552	1.11 (0.83–1.49)	0.489	—
C vs. A	3361	552	1.28 (0.95–1.74)	0.106	—
D vs. A	3361	552	1.25 (0.96–1.64)	0.099	—

*Note:* Cause‐specific survey‐weighted Cox models censored non‐cardiovascular deaths and otherwise followed Table 3. In Stages 2–4, B/C/D versus A estimates came from one four‐level phenotype model. Stage 1 comparisons and Stage 0 were unsupported or structurally non‐estimable as stated. These estimates are not Fine–Gray subdistribution hazard ratios. The corresponding forest plot is Figure S3.

Abbreviations: CI, confidence interval; CKM, cardiovascular–kidney–metabolic; HR, hazard ratio; MASLD, metabolic dysfunction‐associated steatotic liver disease; MI, multiple imputation; NFS, NAFLD fibrosis score; NHANES, National Health and Nutrition Examination Survey.

Direct within‐NFS contrasts addressed whether MASLD status differentiated risk at a fixed NFS category (Table 5; Table S6). Across Stages 2–4, D versus C was not statistically significant for all‐cause mortality (HRs 0.87, 0.87, and 1.14) or cardiovascular mortality (1.50, 1.38, and 1.04). B‐versus‐A estimates were likewise close to the null in the supported stages. Accordingly, below‐null point estimates were not interpreted as a protective MASLD effect.

**TABLE 5:** Direct within‐NFS‐stratum MASLD contrasts for all‐cause and cardiovascular mortality, NHANES 1999–2018.

CKM stage	Contrast	No.	Deaths	Adjusted HR (95% CI)	*p*	Reason if not estimable
All‐cause mortality						
Stage 0	B vs. A	1767	50	Not estimable	—	Sparse contrast cell(s); minimum required per group: *n* ≥ 30 and events ≥ 5. Observed A *n* = 1767/events = 50; B *n* = 0/events = 0
Stage 0	D vs. C	1	0	Not estimable	—	Sparse contrast cell(s); minimum required per group: *n* ≥ 30 and events ≥ 5. Observed C *n* = 1/events = 0; D *n* = 0/events = 0
Stage 1	B vs. A	2791	74	0.99 (0.49–1.99)	0.978	—
Stage 1	D vs. C	15	6	Not estimable	—	Sparse contrast cell(s); minimum required per group: *n* ≥ 30 and events ≥ 5. Observed C *n* = 3/events = 2; D *n* = 12/events = 4
Stage 2	B vs. A	20,944	2151	0.97 (0.87–1.07)	0.525	—
Stage 2	D vs. C	1618	390	0.87 (0.64–1.17)	0.354	—
Stage 3	B vs. A	594	201	0.96 (0.71–1.31)	0.813	—
Stage 3	D vs. C	226	103	0.87 (0.44–1.72)	0.688	—
Stage 4	B vs. A	2478	931	1.14 (0.95–1.38)	0.151	—
Stage 4	D vs. C	883	378	1.14 (0.85–1.53)	0.390	—
Cardiovascular mortality						
Stage 0	B vs. A	1767	10	Not estimable	—	Sparse contrast cell(s); minimum required per group: *n* ≥ 30 and events ≥ 5. Observed A *n* = 1767/events = 10; B *n* = 0/events = 0
Stage 0	D vs. C	1	0	Not estimable	—	Sparse contrast cell(s); minimum required per group: *n* ≥ 30 and events ≥ 5. Observed C *n* = 1/events = 0; D *n* = 0/events = 0
Stage 1	B vs. A	2791	15	Not estimable	—	Sparse contrast cell(s); minimum required per group: *n* ≥ 30 and events ≥ 5. Observed A *n* = 2163/events = 12; B *n* = 628/events = 3
Stage 1	D vs. C	15	2	Not estimable	—	Sparse contrast cell(s); minimum required per group: *n* ≥ 30 and events ≥ 5. Observed C *n* = 3/events = 1; D *n* = 12/events = 1
Stage 2	B vs. A	20,944	617	0.99 (0.82–1.21)	0.937	—
Stage 2	D vs. C	1618	131	1.50 (0.95–2.37)	0.081	—
Stage 3	B vs. A	594	58	0.73 (0.41–1.29)	0.282	—
Stage 3	D vs. C	226	32	1.38 (0.45–4.25)	0.573	—
Stage 4	B vs. A	2478	385	1.09 (0.81–1.47)	0.566	—
Stage 4	D vs. C	883	167	1.04 (0.74–1.47)	0.818	—

*Note:* B versus A compares MASLD status within NFS‐low; D versus C compares MASLD status within NFS‐high. Sparse comparisons remain explicitly non‐estimable. Models used the primary adjustment set and Rubin‐pooled survey covariance. The corresponding forest plot is Figure S4; main Figure 6 addresses the separate exploratory D‐versus‐A subgroup analysis in CKM Stages 2–4.

Abbreviations: CI, confidence interval; CKM, cardiovascular–kidney–metabolic; HR, hazard ratio; MASLD, metabolic dysfunction‐associated steatotic liver disease; MI, multiple imputation; NFS, NAFLD fibrosis score; NHANES, National Health and Nutrition Examination Survey.

### 3.5 Restricted Cubic Spline Analysis of the NAFLD Fibrosis Score

**FIGURE 5:**
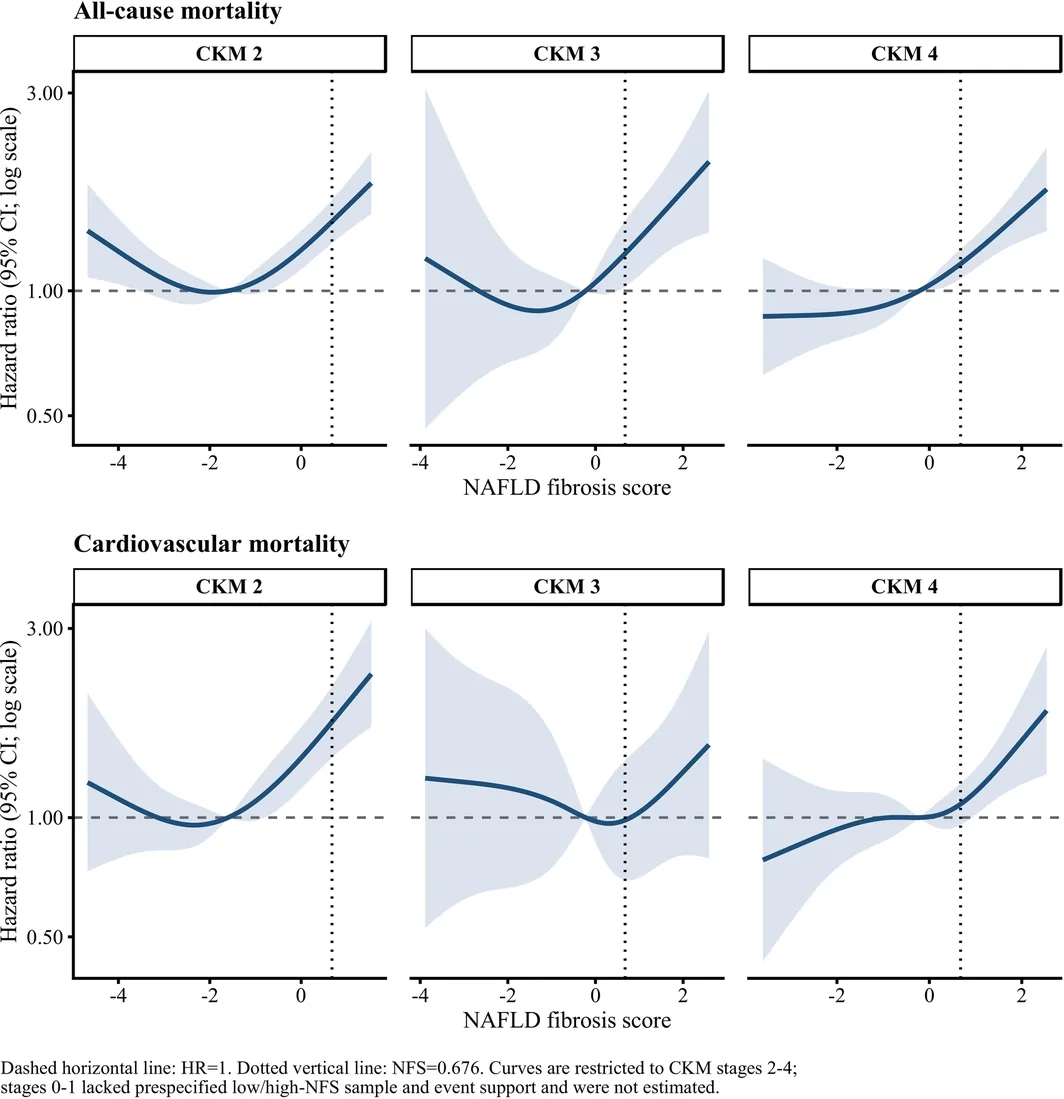
Adjusted restricted‐cubic‐spline associations of continuous NFS with all‐cause mortality (upper panel) and cardiovascular mortality (lower panel) in operational CKM Stages 2–4.

Adjusted NFS spline curves were estimable only in CKM Stages 2–4 (Figure 5; Table S11). Relative to the within‐stage median, higher NFS values generally corresponded to increasing all‐cause and cardiovascular hazard, but low‐score curvature varied by stage and confidence intervals widened towards the distributional tails. The Stages 0–1 panels were suppressed in full because the prespecified NFS‐low/high support rule was not met. No stage‐specific non‐linearity *p* value or common threshold effect was inferred from the displayed curves.

### 3.6 Incremental Predictive Value

The apparent weighted Harrell *C* statistic was 0.8563 for the base model, 0.8566 after adding MASLD, 0.8580 after adding continuous NFS, and 0.8583 after adding the joint phenotype (Table 6; Table S12; Figure S4). Thus, the absolute in‐sample change for the joint phenotype was +0.0020. Pseudo‐likelihood diagnostics also changed, but they were descriptive across imputations and were not a Rubin‐pooled or fully design‐based model‐comparison test. No external validation, calibration, time‐dependent discrimination, reclassification, or net‐benefit result was produced.

**TABLE 6:** Apparent incremental prediction performance for all‐cause mortality in the overall analytic cohort, NHANES 1999–2018 (*N* = 31,317).

Model	Harrell *C* (95% CI)	Δ*C* vs. base	Pseudo AIC, mean (range)	Pseudo LRT *χ* ^2^, mean (range)	Median *p*	No.	Deaths
Base	0.8563 (0.8467–0.8659)	0.0000	52,940.135 (52,929.355–52,949.980)	—	—	31,317	4284
+MASLD	0.8566 (0.8471–0.8662)	+0.0004	52,927.731 (52,916.840–52,937.463)	14.404 (13.547–14.853)	< 0.001	31,317	4284
+continuous NFS	0.8580 (0.8484–0.8677)	+0.0018	52,867.653 (52,856.929–52,877.075)	74.481 (73.126–75.621)	< 0.001	31,317	4284
+joint phenotype	0.8583 (0.8487–0.8679)	+0.0020	52,852.875 (52,841.688–52,863.051)	93.260 (91.811–94.294)	< 0.001	31,317	4284

*Note:* The base model included age, sex, race or ethnicity, educational attainment, family income‐to‐poverty ratio, and smoking. Harrell *C* values are apparent in‐sample estimates with cluster‐robust confidence intervals averaged across imputations. Pseudo AIC and pseudo‐LRT entries show the mean statistic and across‐imputation range; pseudo‐LRT probabilities are descriptive and not Rubin pooled. No external validation, calibration, time‐dependent AUC, NRI, IDI, Brier score, decision‐curve analysis, or net‐benefit analysis was performed. Changes in apparent discrimination must not be interpreted as demonstrated clinical utility. Table S10 and Figure S5 show the Harrell *C* comparison.

Abbreviations: AIC, Akaike information criterion; CI, confidence interval; IDI, integrated discrimination improvement; LRT, likelihood‐ratio test; MASLD, metabolic dysfunction‐associated steatotic liver disease; NFS, NAFLD fibrosis score; NHANES, National Health and Nutrition Examination Survey; NRI, net reclassification improvement.

### 3.7 Subgroup Analyses

**FIGURE 6:**
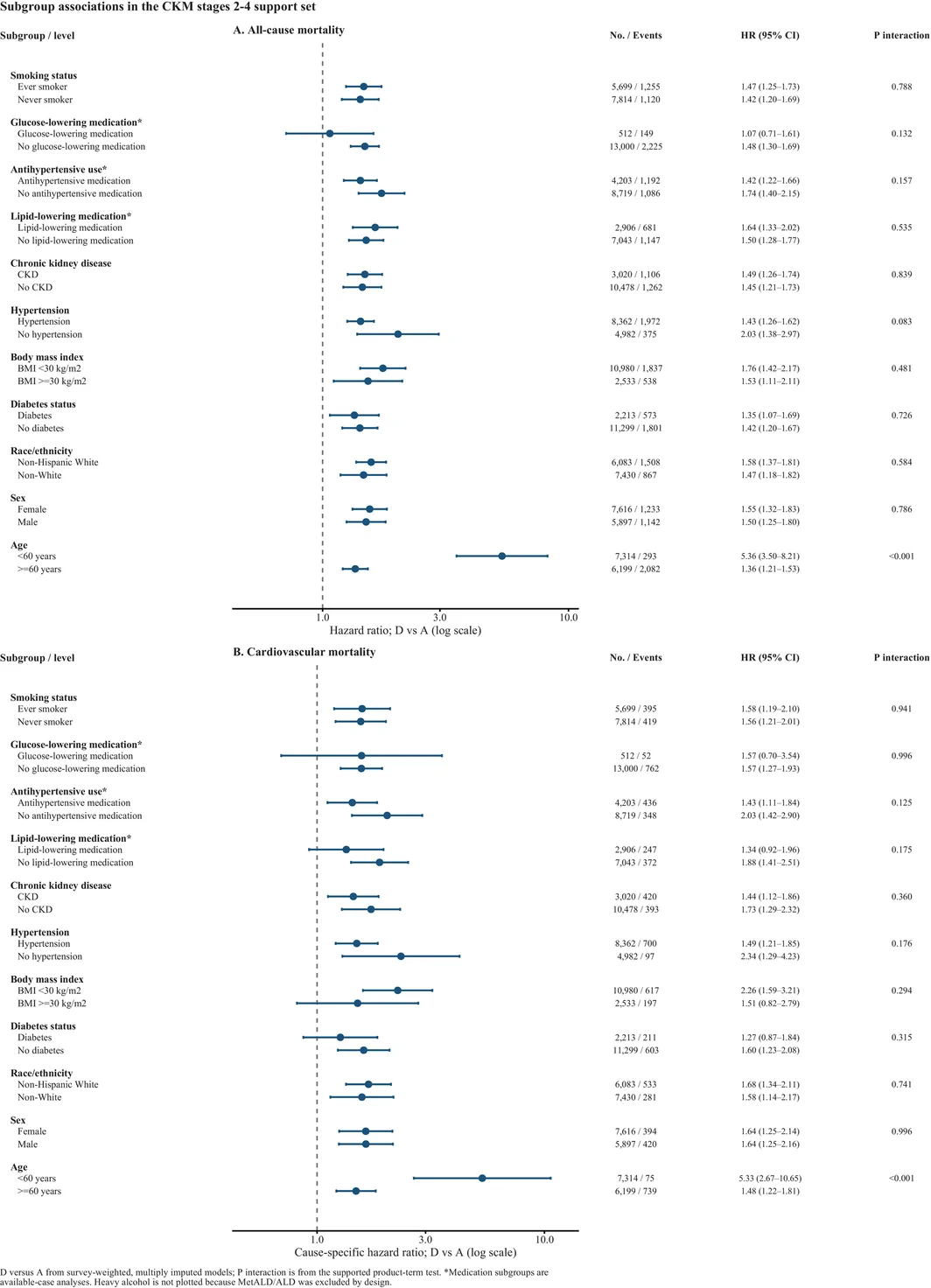
Exploratory D‐versus‐A subgroup associations within the CKM Stages 2–4 support set for all‐cause mortality (panel A) and cause‐specific cardiovascular mortality (panel B).

Exploratory subgroup analyses compared D with A in the CKM Stages 2–4 support set (Figure 6). Age was the only dimension with a nominal interaction *p* value below 0.05 for either outcome. For all‐cause mortality, D versus A was HR 5.36 (95% CI 3.50–8.21) in participants aged < 60 years and 1.36 (1.21–1.53) in those aged ≥ 60 years (*p* interaction = 5.81 × 10^−10^). Corresponding cardiovascular estimates were 5.33 (2.67–10.65) and 1.48 (1.22–1.81; *p* interaction = 0.000602). The other 10 interaction tests for each outcome had *p* ≥ 0.05.

All 11 subgroup dimensions were estimable in both outcome analyses, but interaction tests were nominal and not multiplicity‐adjusted. Medication analyses were available‐case: lipid‐lowering medication status was available for 19,509 participants and missing for 7234; antihypertensive status was available for 25,329 and missing for 1414; and glucose‐lowering status was available for 26,740 and missing for 3. Heavy alcohol was absent by design and no alcohol subgroup hazard ratio was fitted.

### 3.8 Sensitivity Analyses

Sensitivity analyses retained an explicit audit trail rather than replacing non‐estimable primary cells (Figures S2 and S3; Tables S7–S10 and S18). Each additional CMRF was analyzed among 27,027 participants (4290 had missing CMRF count) and was associated with HR 1.05 (95% CI 1.02–1.09; *p* = 0.004) for all‐cause and 1.15 (1.07–1.24; *p* < 0.001) for cardiovascular mortality; phenotype‐by‐CMRF‐group interaction *p* values were 0.188 and 0.088, respectively. Complete‐case, 2‐year landmark, HSI, FIB‐4, fasting‐FLI, and E‐value outputs were reported with their estimability and approximation limits. Twenty‐imputation integrity checks confirmed 31,317 participant identifiers, 626,340 completed‐data rows, complete target‐covariate imputation, and identity of protected fields across all data sets.

Alcohol audits used two deliberately different denominators. In the sequential flow, 8006 participants were excluded for unknown intake and 2329 for MetALD/ALD‐range intake before later phenotype and CKM‐stage exclusions. In an eligible‐except‐alcohol audit that applied all other final criteria while leaving alcohol unfiltered, 1484 were MetALD‐range, 501 ALD‐range, and 6158 unknown. These counts are not additive or interchangeable (Table S16). Liver‐related mortality remained non‐identifiable from public UCOD_LEADING categories (Table S17).

In the reliable 2017–2018 VCTE subset, FLI ≥ 60 had weighted sensitivity 0.717 and specificity 0.772 for CAP ≥ 248 dB/m; unweighted area under the curve was 0.828 (95% CI 0.814–0.842; *n* = 3430). NFS against liver stiffness ≥ 12 kPa had unweighted area under the curve 0.772. The CAP‐defined joint phenotype included 3429 participants and only 42 all‐cause deaths; its four‐level mortality model was not estimable. The retained binary CAP‐MASLD estimate was HR 1.39 (0.63–3.06; *p* = 0.414), and was explicitly exploratory because only one cycle and short follow‐up were available (Tables S13–S15; Figures S5 and S6).

## 4 Discussion

In this rebuilt cohort of 31,317 U.S. adults, four principal findings emerged. First, the corrected participant flow, early‐cycle weight construction, and source‐variable harmonization materially changed the cohort and all downstream estimates. Second, survey‐weighted all‐cause survival and descriptive cardiovascular cumulative‐incidence curves separated across phenotypes in supported CKM stages, but these unadjusted displays answered a different question from the adjusted Cox models. Third, D versus A was associated with higher adjusted all‐cause mortality in Stages 2–4 and with higher cardiovascular mortality in Stage 2, whereas the full Stages 0–4 interaction was structurally non‐estimable and the support‐set interaction tests were not statistically significant. Fourth, apparent prediction gain was small and the exploratory subgroup signal was concentrated in age, without multiplicity adjustment.

Prior observational cohorts and meta‐analyses have linked steatotic liver disease and fibrosis markers to cardiovascular events and mortality [5, 6, 7, 8, 9, 10, 11, 12]. Our results are compatible with the broader proposition that advanced metabolic and fibrosis‐proxy phenotypes identify populations with substantial adverse outcomes, but they also illustrate why an overall association cannot simply be projected onto every CKM stage. Operational CKM stage and NFS share age‐, adiposity‐, glycaemia‐, and kidney‐related information, while FLI depends on adiposity, triglycerides, gamma‐glutamyl transferase, and waist circumference. The resulting construct overlap, together with sparse early‐stage cells, limits claims that either liver score adds an independent biological dimension [15, 16, 17, 18].

The distinction between descriptive and adjusted estimands is central. Survey‐weighted Kaplan–Meier curves describe population survival experience without covariate adjustment; their separation does not establish causation or protection. Weighted Aalen–Johansen curves describe cardiovascular cumulative incidence while accounting for competing non‐cardiovascular death, but the present implementation does not supply a design‐based Gray test, covariate‐adjusted Fine–Gray model, or subdistribution hazard ratio. Accordingly, adjusted interpretation rests on cause‐specific survey Cox estimates and their confidence intervals, with the curves retained as complementary absolute‐risk descriptions.

The advanced‐stage results do not support the earlier interpretation of a protective or biologically saturated MASLD effect. In Stage 4, D versus A remained associated with all‐cause mortality, whereas the cardiovascular estimate was imprecise and included the null; direct D‐versus‐C estimates were also compatible with no difference. Conditioning on advanced CKM can change the composition of comparison groups through high baseline risk, treatment, survival, and selection. Group C is especially heterogeneous: its members were older and had lower kidney function, and a low FLI can occur despite previous or advanced liver disease. Regression of steatosis or so‐called burned‐out MASLD is one possible explanation, but a single cross‐sectional FLI/NFS assessment cannot establish that history.

The age interaction is hypothesis‐generating rather than a clinical targeting result. Younger participants had larger relative D‐versus‐A hazard ratios, but their baseline event risk, selection into the supported phenotypes, and residual differences may not match those of older adults. Ten other subgroup interactions were non‐significant for each outcome, and no multiplicity correction was applied. Medication subgroups were available‐case and remain susceptible to missingness, confounding by indication, treatment access, and reverse causation. The source category is lipid‐lowering medication use and cannot be narrowed to statins.

Clinically, the +0.0020 apparent *C*‐statistic change is too small and too internally assessed to establish usefulness [25, 26]. The absence of external validation, calibration at clinical horizons, decision‐curve analysis, and treatment‐impact evaluation precludes a recommendation for routine phenotype‐guided care. The VCTE analysis reinforces this restraint: FLI and NFS showed measurable cross‐sectional discrimination against CAP and liver‐stiffness thresholds, but agreement was incomplete and the CAP‐defined mortality cohort had only 42 deaths. These proxies may be useful for research stratification, but imaging, histology, and clinical decision pathways address different purposes [16, 22, 23, 28, 29].

Strengths include reconstruction from original NHANES component files, a participant‐level exclusion log, correction of the 20‐year survey‐weight formula, inclusion of all 10 cycles, cycle‐nested strata and primary sampling units, covariate‐only multiple imputation, separation of descriptive and adjusted estimands, competing‐death accounting in the descriptive cardiovascular curves, and explicit sparse‐cell suppression. Limitations include the retrospective observational design; single‐time‐point FLI and NFS; proxy and self‐report misclassification; substitution of a harmonized diabetes/HbA1c indicator in NFS; an operational rather than complete AHA CKM Stage 3 implementation; limited covariate harmonization over 20 years; residual confounding and selection; available‐case medication subgroups; nominal exploratory interactions; apparent rather than validated prediction; and VCTE availability in one cycle. Public‐use mortality categories also prevented identification of liver‐related death.

## 5 Conclusions

In this retrospective analysis of 31,317 NHANES adults, joint FLI‐defined MASLD/NFS phenotypes showed heterogeneous descriptive outcome patterns and adjusted mortality associations across supported operational CKM stages. The complete Stages 0–4 interaction was structurally unsupported, below‐null contrasts did not demonstrate protection, and the age subgroup interaction was exploratory. Together with the small, unvalidated apparent prediction change, these results support cautious independent validation of liver‐proxy phenotyping within CKM research rather than immediate clinical implementation.

## Ethics Statement

NHANES protocols were approved by the National Center for Health Statistics (NCHS) Research Ethics Review Board, and all participants provided written informed consent. The present analysis used fully de‐identified, publicly available data and was therefore exempt from additional institutional review board review.

## Conflicts of Interest

The authors declare no conflicts of interest.

## Supporting information


**Table S1:** Sequential participant exclusions from the 10‐cycle NHANES source population.
**Table S2:** Cycle‐specific construction and audit of the pooled 20‐year survey weights.
**Table S3:** Detailed survey‐weighted characteristics by operational CKM stage.
**Table S4:** Detailed survey‐weighted characteristics by joint MASLD–NFS phenotype.
**Table S5:** Survey‐weighted joint‐phenotype prevalence by NHANES cycle and operational CKM stage.
**Table S6:** Kaplan–Meier support, landmark risk sets, survival estimates, and design‐based log‐rank tests.
**Table S7:** Weighted Aalen–Johansen cardiovascular cumulative‐incidence support and landmark risk sets.
**Table S8:** Joint‐phenotype interaction tests for CKM stage and cardiometabolic risk‐factor burden.
**Table S9:** Restricted‐cubic‐spline support, modeling range, reference, and knot audit.
**Table S10:** Apparent weighted Harrell *C* and incremental in‐sample discrimination.
**Table S11:** Exploratory D‐versus‐A subgroup effects, interaction tests, and availability.
**Table S12:** Cardiometabolic risk‐factor burden and mortality analyses.
**Table S13:** Prespecified mortality sensitivity analyses.
**Table S14:** Multiple‐imputation targets and completed‐data integrity.
**Table S15:** Alcohol‐etiology category audit in the eligible‐except‐alcohol population.
**Table S16:** Feasibility of liver‐related mortality ascertainment.
**Table S17:** Vibration‐controlled transient‐elastography proxy performance in NHANES 2017–2018.
**Table S18:** CAP‐defined joint‐phenotype counts and exploratory mortality sensitivity analysis.
**Figure S1:** Temporal trends in the joint MASLD–NFS phenotypes. Survey‐weighted prevalence estimates are shown across the 10 NHANES cycles overall and within operational CKM Stages 0–4. Lines and points denote phenotypes A (no MASLD/NFS‐low), B (MASLD/NFS‐low), C (no MASLD/NFS‐high), and D (MASLD/NFS‐high). Estimates are unadjusted descriptive proportions based on the cycle‐appropriate MEC survey design.
**Figure S2:** Adjusted all‐cause mortality associations by operational CKM stage. Points and horizontal bars show survey‐weighted hazard ratios and 95% confidence intervals combined across 20 covariate‐only imputations. Stages 2–4 use the same fully adjusted four‐level phenotype model with A as reference; Stage 1 retains only the supported NFS‐low A/B comparison, and Stage 0 is structurally unsupported. This adjusted figure complements the unadjusted Kaplan–Meier curves in Figure 3.
**Figure S3:** Adjusted cause‐specific cardiovascular mortality associations by operational CKM stage. Points and horizontal bars show survey‐weighted, multiply imputed cause‐specific Cox hazard ratios and 95% confidence intervals; non‐cardiovascular deaths are censored. Sparse Stages 0–1 contrasts remain explicit in the source data. These estimates are not Fine–Gray subdistribution hazard ratios and complement the descriptive weighted Aalen‐Johansen curves in Figure 4.
**Figure S4:** Direct within‐NFS‐stratum MASLD contrasts for all‐cause and cardiovascular mortality. B versus A compares MASLD status within NFS‐low, and D versus C compares MASLD status within NFS‐high. Points and horizontal bars show survey‐weighted, multiply imputed hazard ratios and 95% confidence intervals. Below‐null estimates are associations and do not establish a protective effect. Main Figure 6 addresses the separate exploratory D‐versus‐A subgroup question.
**Figure S5:** Apparent incremental in‐sample discrimination. Points and horizontal bars show apparent weighted Harrell *C* estimates and 95% confidence intervals for the base, +MASLD, +NFS, and +joint models overall and by operational CKM stage. These are exploratory in‐sample estimates; no external validation, time‐dependent AUC, Brier score, NRI, IDI, or decision‐curve net benefit was estimated.
**Figure S6:** Cardiometabolic risk‐factor burden and mortality. Points and horizontal bars show hazard ratios and 95% confidence intervals on a logarithmic scale for all‐cause and cardiovascular mortality. The figure includes the continuous effect per one additional cardiometabolic risk factor and prespecified B‐versus‐A and D‐versus‐C contrasts. Models use the multiply imputed survey analysis and prespecified adjustment covariates; non‐estimable rows and their reasons remain in the source data.
**Figure S7:** Prespecified mortality sensitivity analyses. Survey‐weighted hazard ratios and 95% confidence intervals are displayed by outcome, contrast, and operational CKM stage for the complete‐case, 2‐year landmark, HSI ≥ 36, FIB‐4 ≥ 1.3, and fasting‐triglyceride FLI analyses. The vertical reference line is HR = 1. Non‐estimable stage–contrast combinations are omitted from the plot but retained with explicit reasons in the source table.
**Figure S8:** Cross‐sectional agreement with vibration‐controlled transient elastography proxies in the reliable NHANES 2017–2018 subset. Survey‐weighted sensitivity, specificity, positive predictive value, and negative predictive value are shown for FLI ≥ 60 against CAP ≥ 248 dB/m, and for NFS > 0.676 and FIB‐4 ≥ 1.3 against LSM ≥ 8 and LSM ≥ 12 kPa. CAP and LSM are noninvasive proxy reference standards, not biopsy or histological diagnoses.
**Figure S9:** VCTE diagnostic sensitivity analysis. Points and horizontal bars show unweighted receiver‐operating‐characteristic areas under the curve with DeLong 95% confidence intervals for continuous FLI, NFS, and FIB‐4 against CAP ≥ 248 dB/m and LSM ≥ 8 or LSM ≥ 12 kPa. The dashed reference line denotes AUC = 0.5. DeLong intervals do not account for the complex survey design and are not mortality‐prediction metrics.

## Data Availability

NHANES source files and public‐use linked mortality data are available from the National Center for Health Statistics [19, 20, 21].
