# Supplementary material for: Stage‐Dependent Prognostic Value of the Joint MASLD–NAFLD Fibrosis Score Phenotype Across the Cardiovascular–Kidney–Metabolic Syndrome Continuum: A Retrospective NHANES Cohort Analysis, 1999–2018
Source: Kaohsiung J Med Sci. 2026 Aug 26:e70285. Online ahead of print. doi: 10.1002/kjm2.70285 (PMC13519501; doi:10.1002/kjm2.70285)
Supplement: Supplementary file 1 [file KJM2-9999-e70285-s001.docx]

**Supplementary Materials**

**Supplementary Methods**

***Cohort reconstruction and survey design.*** Ten NHANES cycles from 1999–2000 through 2017–2018 were harmonised from the original component files and linked to the public-use 2019 mortality file. Each source participant received one mutually exclusive first-exclusion reason. The primary 20-year MEC weight was WTMEC4YR × 4/20 in 1999–2002 and WTMEC2YR × 2/20 in 2003–2018; masked strata and primary sampling units were nested within cycle. The fasting-FLI sensitivity analysis used the corresponding pooled fasting-subsample weights.

***Multiple imputation and protected fields.*** Twenty chained-equation imputations with 10 iterations were created for education, income-to-poverty ratio, marital status, and smoking. Marital status was an auxiliary imputation variable and was not included in the primary adjustment set. Exposure, phenotype, CKM stage, outcome, follow-up, survey weight, stratum, primary sampling unit, and other analysis-defining fields were not imputed. The completed archive contains 31,317 participants and 626,340 rows, with participant IDs and protected values identical across all 20 data sets.

***Support and estimability rules.*** A displayed phenotype-stage curve or regression contrast required at least 30 participants and five outcome events in each comparison cell. Curve tails were not displayed after the unweighted risk set fell below 10. The complete phenotype-by-stage interaction across stages 0–4 was attempted but was structurally non-estimable; a separate interaction in the common stages 2–4 support set was reported. Continuous NFS splines were likewise restricted to stages 2–4.

***Survival and competing-risk summaries.*** Survey-weighted Kaplan–Meier curves and design-based log-rank tests used the protected 20-year survey fields once. Cardiovascular cumulative incidence used a descriptive 20-year MEC-weighted Aalen–Johansen estimator with non-cardiovascular death as a competing event. The latter analysis did not generate a design-based Gray test, Fine–Gray model, subdistribution hazard ratio, inferential P value, or design-based confidence interval.

***Sensitivity and validation analyses.*** Prespecified analyses included complete-case and 2-year landmark survey Cox models, alternative HSI and FIB-4 definitions, fasting-triglyceride FLI, CMRF burden, alcohol-aetiology classification, and VCTE proxy comparisons. Medication subgroups were observed-status available-case analyses. VCTE analyses were limited to 2017–2018 and used CAP and LSM as non-invasive proxy standards rather than histological diagnoses.

**Supplementary Table S1. Sequential participant exclusions from the ten-cycle NHANES source population**

| **Step** | **Mutually exclusive first-exclusion reason** | **Excluded at step** | **Remaining** |
| --- | --- | --- | --- |
| 0 | Source NHANES participants (10 cycles) | 0 | 101,316 |
| 1 | Age <20 years or age missing | 46,235 | 55,081 |
| 2 | Not MEC examined | 2,683 | 52,398 |
| 3 | Pregnant at examination | 1,498 | 50,900 |
| 4 | Mortality ineligible or follow-up missing/nonpositive | 121 | 50,779 |
| 5 | Viral-hepatitis status unknown | 3,211 | 47,568 |
| 6 | HBsAg or HCV positive | 954 | 46,614 |
| 7 | ALT or AST >500 U/L | 11 | 46,603 |
| 8 | Alcohol exposure unknown | 8,006 | 38,597 |
| 9 | MetALD or ALD alcohol range | 2,329 | 36,268 |
| 10 | FLI/NFS joint phenotype unclassifiable | 1,571 | 34,697 |
| 11 | Operational CKM stage unclassifiable | 3,380 | 31,317 |
| 12 | Survey design/20-year weight missing or nonpositive | 0 | 31,317 |
| 13 | Final analytic cohort | 0 | 31,317 |

***Note.*** *Each source participant was assigned one mutually exclusive first-exclusion reason. ALT, alanine aminotransferase; AST, aspartate aminotransferase; CKM, cardiovascular–kidney–metabolic; FLI, fatty liver index; MEC, mobile examination centre; NFS, NAFLD fibrosis score.*

**Supplementary Table S2. Cycle-specific construction and audit of the pooled 20-year survey weights**

| **NHANES cycle** | **Weight source** | **20-year formula** | **N** | **Pooled weight min / median / max** | **Pooled weight sum** | **Maximum absolute formula error** | **Fasting N** |
| --- | --- | --- | --- | --- | --- | --- | --- |
| 1999–2000 | WTMEC4YR | WTMEC4YR × 4/20 | 2,546 | 147.60 / 3,478.38 / 20,766.23 | 11,481,874.84 | 3.64e-12 | 1,141 |
| 2001–2002 | WTMEC4YR | WTMEC4YR × 4/20 | 2,740 | 199.21 / 3,609.24 / 20,766.23 | 12,264,854.98 | 1.82e-12 | 0 |
| 2003–2004 | WTMEC2YR | WTMEC2YR × 2/20 | 2,519 | 214.48 / 3,507.52 / 14,584.36 | 11,521,298.79 | 1.82e-12 | 1,108 |
| 2005–2006 | WTMEC2YR | WTMEC2YR × 2/20 | 2,569 | 143.02 / 3,501.43 / 15,615.22 | 12,244,613.29 | 1.82e-12 | 1,119 |
| 2007–2008 | WTMEC2YR | WTMEC2YR × 2/20 | 3,318 | 277.83 / 2,716.54 / 16,085.78 | 12,834,436.77 | 1.82e-12 | 0 |
| 2009–2010 | WTMEC2YR | WTMEC2YR × 2/20 | 3,427 | 429.18 / 2,634.67 / 15,814.69 | 12,760,778.82 | 1.82e-12 | 1,605 |
| 2011–2012 | WTMEC2YR | WTMEC2YR × 2/20 | 3,218 | 441.33 / 2,677.45 / 22,257.98 | 14,282,376.39 | 3.64e-12 | 1,495 |
| 2013–2014 | WTMEC2YR | WTMEC2YR × 2/20 | 3,648 | 575.17 / 2,952.46 / 17,107.52 | 15,570,648.12 | 1.82e-12 | 1,655 |
| 2015–2016 | WTMEC2YR | WTMEC2YR × 2/20 | 3,510 | 547.00 / 2,681.25 / 23,029.66 | 15,585,606.49 | 3.64e-12 | 1,558 |
| 2017–2018 | WTMEC2YR | WTMEC2YR × 2/20 | 3,822 | 464.50 / 2,731.83 / 41,976.28 | 17,968,687.76 | 7.28e-12 | 1,745 |

***Note.*** *The 1999–2002 four-year MEC weight was divided across the full 20-year interval as WTMEC4YR × 4/20; each 2003–2018 two-year MEC weight used WTMEC2YR × 2/20. Small non-zero formula differences reflect floating-point precision. MEC, mobile examination centre.*

**Supplementary Table S3. Detailed survey-weighted characteristics by operational CKM stage**

| **Characteristic or category** | **Stage 0** | **Stage 1** | **Stage 2** | **Stage 3** | **Stage 4** |
| --- | --- | --- | --- | --- | --- |
| Age, years | 35.90 (35.09–36.71) | 39.99 (39.29–40.68) | 48.08 (47.69–48.46) | 62.55 (60.78–64.31) | 64.64 (63.99–65.29) |
| Body mass index, kg/m² | 21.56 (21.44–21.68) | 28.01 (27.78–28.23) | 29.88 (29.72–30.04) | 30.53 (29.86–31.20) | 30.07 (29.78–30.37) |
| Waist circumference, cm | 78.30 (77.93–78.66) | 95.13 (94.65–95.62) | 101.20 (100.81–101.59) | 104.63 (102.97–106.30) | 104.78 (104.07–105.50) |
| Systolic blood pressure, mmHg | 110.38 (109.73–111.02) | 112.04 (111.67–112.42) | 125.13 (124.76–125.49) | 138.26 (135.77–140.76) | 131.00 (130.02–131.99) |
| Diastolic blood pressure, mmHg | 67.19 (66.65–67.72) | 68.54 (68.09–68.99) | 72.81 (72.47–73.14) | 68.46 (66.68–70.25) | 67.81 (67.10–68.51) |
| Glycated haemoglobin, % | 5.14 (5.12–5.16) | 5.19 (5.18–5.20) | 5.65 (5.63–5.66) | 6.49 (6.31–6.67) | 6.02 (5.98–6.07) |
| Triglycerides, mg/dL | 74.75 (73.24–76.26) | 83.91 (82.66–85.17) | 171.61 (168.80–174.42) | 195.36 (176.68–214.04) | 167.06 (161.60–172.52) |
| HDL cholesterol, mg/dL | 63.74 (62.90–64.59) | 61.58 (60.91–62.25) | 49.54 (49.23–49.86) | 51.61 (49.93–53.29) | 50.41 (49.60–51.22) |
| Total cholesterol, mg/dL | 178.19 (176.24–180.13) | 188.19 (186.49–189.90) | 200.33 (199.42–201.25) | 198.56 (193.70–203.42) | 187.19 (184.78–189.60) |
| Estimated glomerular filtration rate, mL/min/1.73 m² | 105.81 (104.79–106.84) | 102.59 (101.67–103.50) | 96.64 (96.13–97.14) | 59.47 (55.96–62.97) | 77.53 (76.49–78.56) |
| Urine albumin-to-creatinine ratio, mg/g | 7.22 (6.95–7.49) | 6.70 (6.48–6.92) | 14.32 (13.80–14.84) | 673.50 (572.72–774.27) | 84.04 (66.80–101.27) |
| Fatty liver index | 9.02 (8.54–9.51) | 37.36 (36.22–38.50) | 59.69 (59.01–60.37) | 63.02 (60.14–65.91) | 63.90 (62.51–65.30) |
| NAFLD fibrosis score | -3.15 (-3.20–-3.09) | -2.51 (-2.56–-2.45) | -1.83 (-1.86–-1.79) | -0.50 (-0.68–-0.33) | -0.47 (-0.54–-0.40) |
| FIB-4 | 0.84 (0.81–0.87) | 0.87 (0.84–0.90) | 1.02 (1.00–1.03) | 1.52 (1.44–1.60) | 1.64 (1.59–1.68) |
| Hepatic steatosis index | 29.46 (29.27–29.64) | 36.86 (36.61–37.11) | 39.44 (39.25–39.64) | 39.62 (38.85–40.39) | 39.12 (38.76–39.47) |
| Cardiometabolic risk-factor count | 0.00 (0.00–0.00) | 1.00 (1.00–1.00) | 2.88 (2.85–2.90) | 3.57 (3.45–3.68) | 3.49 (3.43–3.55) |
| Alcohol consumption, g/day | 5.29 (4.83–5.75) | 4.83 (4.49–5.16) | 4.14 (3.98–4.30) | 2.37 (1.86–2.88) | 3.34 (3.04–3.64) |
| Sex: Male | 48.18% (45.19–51.17) | 35.94% (33.73–38.15) | 48.52% (47.70–49.34) | 38.61% (34.05–43.17) | 51.64% (49.21–54.07) |
| Sex: Female | 51.82% (48.83–54.81) | 64.06% (61.85–66.27) | 51.48% (50.66–52.30) | 61.39% (56.83–65.95) | 48.36% (45.93–50.79) |
| Race or ethnicity: Mexican American | 5.23% (4.15–6.31) | 7.59% (6.23–8.95) | 8.67% (7.51–9.83) | 8.64% (6.35–10.92) | 4.08% (3.21–4.95) |
| Race or ethnicity: Non-Hispanic Black | 7.79% (6.60–8.97) | 9.10% (7.71–10.49) | 10.47% (9.31–11.62) | 17.63% (14.41–20.84) | 10.18% (8.86–11.51) |
| Race or ethnicity: Non-Hispanic White | 73.40% (70.94–75.86) | 72.94% (70.09–75.79) | 68.23% (66.05–70.41) | 59.63% (55.19–64.07) | 76.57% (74.31–78.83) |
| Race or ethnicity: Other Hispanic | 5.11% (3.80–6.43) | 5.83% (4.59–7.07) | 5.89% (4.95–6.83) | 6.18% (3.84–8.51) | 3.52% (2.59–4.46) |
| Race or ethnicity: Other/multiracial | 8.47% (7.10–9.83) | 4.55% (3.42–5.68) | 6.74% (6.08–7.40) | 7.93% (5.27–10.58) | 5.64% (4.38–6.89) |
| Education: 9th-11th grade | 5.53% (4.34–6.73) | 5.68% (4.63–6.74) | 10.59% (9.92–11.27) | 14.28% (11.48–17.08) | 14.61% (13.19–16.04) |
| Education: College graduate or above | 47.30% (43.63–50.96) | 44.03% (41.20–46.86) | 27.58% (26.09–29.07) | 18.84% (14.83–22.86) | 19.72% (17.71–21.74) |
| Education: High school/GED | 17.05% (14.49–19.61) | 16.39% (14.60–18.17) | 24.35% (23.29–25.40) | 26.18% (22.14–30.21) | 27.19% (25.06–29.33) |
| Education: Less than 9th grade | 1.02% (0.56–1.48) | 2.13% (1.56–2.70) | 5.47% (5.00–5.94) | 12.32% (9.62–15.03) | 8.91% (7.71–10.10) |
| Education: Some college/AA | 29.10% (26.04–32.16) | 31.77% (29.63–33.91) | 32.01% (31.07–32.96) | 28.38% (23.87–32.88) | 29.56% (27.50–31.62) |
| Smoking status: Never | 67.56% (64.91–70.20) | 64.19% (62.11–66.28) | 56.98% (55.79–58.16) | 54.06% (49.60–58.52) | 43.07% (40.57–45.56) |
| Smoking status: Former | 15.27% (13.05–17.48) | 22.48% (20.54–24.41) | 23.62% (22.77–24.47) | 31.53% (27.35–35.71) | 37.36% (35.24–39.48) |
| Smoking status: Current | 17.18% (15.02–19.34) | 13.33% (11.60–15.06) | 19.40% (18.48–20.32) | 14.41% (10.89–17.94) | 19.57% (17.76–21.38) |
| Hypertension: No | 94.34% (92.98–95.70) | 90.83% (89.51–92.14) | 40.56% (39.57–41.55) | 15.51% (12.21–18.82) | 19.37% (17.27–21.47) |
| Hypertension: Yes | 5.66% (4.30–7.02) | 9.17% (7.86–10.49) | 59.44% (58.45–60.43) | 84.49% (81.18–87.79) | 80.63% (78.53–82.73) |
| Diabetes: No | 100.00% (100.00–100.00) | 100.00% (100.00–100.00) | 88.56% (88.02–89.10) | 61.86% (56.75–66.96) | 70.35% (68.45–72.26) |
| Diabetes: Yes | 0.00% (0.00–0.00) | 0.00% (0.00–0.00) | 11.44% (10.90–11.98) | 38.14% (33.04–43.25) | 29.65% (27.74–31.55) |
| Prediabetes: No | 100.00% (100.00–100.00) | 100.00% (100.00–100.00) | 75.89% (75.21–76.57) | 76.85% (72.47–81.23) | 72.63% (70.62–74.64) |
| Prediabetes: Yes | 0.00% (0.00–0.00) | 0.00% (0.00–0.00) | 24.11% (23.43–24.79) | 23.15% (18.77–27.53) | 27.37% (25.36–29.38) |
| Joint phenotype: A | 99.98% (99.94–100.00) | 77.94% (76.02–79.86) | 44.58% (43.56–45.59) | 34.33% (29.90–38.75) | 34.69% (32.45–36.92) |
| Joint phenotype: B | 0.00% (0.00–0.00) | 21.69% (19.79–23.58) | 50.29% (49.32–51.26) | 40.91% (36.99–44.83) | 41.48% (39.14–43.81) |
| Joint phenotype: C | 0.02% (0.00–0.06) | 0.07% (0.00–0.15) | 0.89% (0.77–1.01) | 6.17% (4.00–8.33) | 5.46% (4.51–6.41) |
| Joint phenotype: D | 0.00% (0.00–0.00) | 0.31% (0.12–0.49) | 4.24% (3.91–4.57) | 18.59% (14.95–22.23) | 18.38% (16.75–20.01) |

***Note.*** *Values are survey-weighted means or percentages with design-based 95% confidence intervals; sample counts are unweighted. These detailed estimates complement the selected characteristics in main Table 1. CKM, cardiovascular–kidney–metabolic; FIB-4, fibrosis-4 index; FLI, fatty liver index; HDL, high-density lipoprotein; HSI, hepatic steatosis index; NFS, NAFLD fibrosis score.*

**Supplementary Table S4. Detailed survey-weighted characteristics by joint MASLD–NFS phenotype**

| **Characteristic or category** | **A** | **B** | **C** | **D** |
| --- | --- | --- | --- | --- |
| Age, years | 45.65 (45.15–46.16) | 47.82 (47.43–48.21) | 75.43 (74.58–76.27) | 65.93 (65.13–66.73) |
| Body mass index, kg/m² | 24.71 (24.63–24.78) | 33.43 (33.27–33.60) | 26.37 (26.05–26.69) | 38.23 (37.74–38.72) |
| Waist circumference, cm | 87.82 (87.59–88.05) | 110.49 (110.14–110.83) | 95.63 (94.83–96.43) | 122.13 (121.17–123.09) |
| Systolic blood pressure, mmHg | 119.91 (119.47–120.35) | 125.71 (125.34–126.07) | 137.18 (134.27–140.09) | 133.90 (132.82–134.97) |
| Diastolic blood pressure, mmHg | 69.76 (69.41–70.10) | 74.01 (73.63–74.39) | 62.93 (61.52–64.35) | 68.29 (67.42–69.17) |
| Glycated haemoglobin, % | 5.39 (5.37–5.40) | 5.75 (5.73–5.77) | 6.08 (6.00–6.15) | 6.50 (6.44–6.55) |
| Triglycerides, mg/dL | 107.95 (106.63–109.27) | 208.98 (204.70–213.27) | 104.80 (100.02–109.59) | 184.60 (174.58–194.62) |
| HDL cholesterol, mg/dL | 57.56 (57.12–58.01) | 45.64 (45.35–45.94) | 58.67 (56.62–60.71) | 48.25 (47.51–49.00) |
| Total cholesterol, mg/dL | 190.89 (190.02–191.76) | 204.37 (203.10–205.63) | 176.72 (172.01–181.43) | 186.25 (183.72–188.78) |
| Estimated glomerular filtration rate, mL/min/1.73 m² | 97.99 (97.40–98.58) | 95.98 (95.42–96.53) | 67.63 (65.43–69.84) | 76.58 (75.12–78.03) |
| Urine albumin-to-creatinine ratio, mg/g | 20.24 (18.28–22.19) | 33.22 (28.72–37.71) | 88.44 (46.96–129.92) | 87.97 (69.47–106.48) |
| Fatty liver index | 26.37 (25.99–26.74) | 83.55 (83.25–83.84) | 36.58 (35.29–37.86) | 89.97 (89.31–90.63) |
| NAFLD fibrosis score | -2.41 (-2.44–-2.37) | -1.67 (-1.70–-1.63) | 1.23 (1.18–1.27) | 1.42 (1.38–1.45) |
| FIB-4 | 1.00 (0.99–1.02) | 0.93 (0.91–0.94) | 2.97 (2.79–3.16) | 2.07 (1.96–2.19) |
| Hepatic steatosis index | 33.22 (33.12–33.31) | 43.78 (43.58–43.97) | 33.80 (33.37–34.23) | 47.43 (46.87–48.00) |
| Cardiometabolic risk-factor count | 1.68 (1.65–1.71) | 3.20 (3.17–3.23) | 3.42 (3.33–3.51) | 4.02 (3.97–4.08) |
| Alcohol consumption, g/day | 4.34 (4.17–4.52) | 4.30 (4.13–4.47) | 2.49 (1.88–3.11) | 2.30 (1.98–2.62) |
| Sex: Male | 40.34% (39.31–41.37) | 55.58% (54.50–56.66) | 44.78% (39.69–49.88) | 45.69% (42.60–48.79) |
| Sex: Female | 59.66% (58.63–60.69) | 44.42% (43.34–45.50) | 55.22% (50.12–60.31) | 54.31% (51.21–57.40) |
| Race or ethnicity: Mexican American | 6.44% (5.58–7.30) | 10.08% (8.64–11.52) | 2.99% (1.74–4.24) | 5.75% (4.41–7.09) |
| Race or ethnicity: Non-Hispanic Black | 9.71% (8.70–10.73) | 10.06% (8.85–11.26) | 14.53% (11.32–17.74) | 15.78% (13.43–18.13) |
| Race or ethnicity: Non-Hispanic White | 70.34% (68.36–72.32) | 68.70% (66.35–71.06) | 71.35% (66.42–76.27) | 71.23% (68.15–74.31) |
| Race or ethnicity: Other Hispanic | 5.66% (4.71–6.61) | 5.90% (4.89–6.91) | 2.96% (1.75–4.16) | 3.71% (2.76–4.67) |
| Race or ethnicity: Other/multiracial | 7.84% (7.00–8.69) | 5.26% (4.66–5.86) | 8.18% (4.97–11.38) | 3.53% (2.33–4.73) |
| Education: 9th-11th grade | 9.25% (8.50–9.99) | 10.73% (10.00–11.46) | 11.69% (8.97–14.42) | 12.57% (10.85–14.29) |
| Education: College graduate or above | 35.04% (33.06–37.01) | 25.35% (23.85–26.86) | 23.31% (18.95–27.67) | 20.45% (17.94–22.96) |
| Education: High school/GED | 21.08% (19.97–22.18) | 25.15% (23.94–26.37) | 26.46% (21.60–31.31) | 27.31% (24.63–29.99) |
| Education: Less than 9th grade | 4.53% (4.07–4.98) | 5.42% (4.84–6.00) | 13.23% (10.54–15.91) | 7.95% (6.59–9.31) |
| Education: Some college/AA | 30.11% (28.90–31.33) | 33.34% (32.18–34.51) | 25.32% (20.64–30.00) | 31.72% (29.10–34.34) |
| Smoking status: Never | 60.07% (58.73–61.42) | 54.60% (53.24–55.97) | 54.91% (50.17–59.65) | 52.80% (49.57–56.03) |
| Smoking status: Former | 20.36% (19.38–21.34) | 26.77% (25.58–27.96) | 38.68% (33.53–43.84) | 38.50% (35.54–41.45) |
| Smoking status: Current | 19.57% (18.47–20.67) | 18.63% (17.70–19.56) | 6.41% (4.29–8.53) | 8.70% (7.23–10.17) |
| Hypertension: No | 61.03% (59.93–62.13) | 37.16% (35.93–38.38) | 13.65% (10.68–16.61) | 14.47% (11.99–16.94) |
| Hypertension: Yes | 38.97% (37.87–40.07) | 62.84% (61.62–64.07) | 86.35% (83.39–89.32) | 85.53% (83.06–88.01) |
| Diabetes: No | 95.30% (94.86–95.75) | 85.73% (84.95–86.50) | 66.21% (61.13–71.30) | 50.88% (48.18–53.58) |
| Diabetes: Yes | 4.70% (4.25–5.14) | 14.27% (13.50–15.05) | 33.79% (28.70–38.87) | 49.12% (46.42–51.82) |
| Prediabetes: No | 85.89% (85.22–86.56) | 76.79% (75.91–77.67) | 45.05% (39.64–50.45) | 57.46% (54.59–60.33) |
| Prediabetes: Yes | 14.11% (13.44–14.78) | 23.21% (22.33–24.09) | 54.95% (49.55–60.36) | 42.54% (39.67–45.41) |

***Note.*** *Values are survey-weighted means or percentages with design-based 95% confidence intervals. A, no MASLD/NFS-low; B, MASLD/NFS-low; C, no MASLD/NFS-high; D, MASLD/NFS-high. NFS-low is an epidemiological score category and not histological absence of fibrosis.*

**Supplementary Table S5. Survey-weighted joint-phenotype prevalence by NHANES cycle and operational CKM stage**

| **Cycle** | **Operational CKM stage** | **Unweighted N** | **A, % (95% CI)** | **B, % (95% CI)** | **C, % (95% CI)** | **D, % (95% CI)** |
| --- | --- | --- | --- | --- | --- | --- |
| 1999–2000 | Overall | 2,546 | 56.5% (51.7–61.3) | 40.7% (36.3–45.1) | 0.5% (0.2–0.9) | 2.3% (1.6–2.9) |
| 1999–2000 | Stage 0 | 122 | 100.0% (100.0–100.0) | 0.0% (0.0–0.0) | 0.0% (0.0–0.0) | 0.0% (0.0–0.0) |
| 1999–2000 | Stage 1 | 166 | 80.6% (74.4–86.8) | 18.9% (13.3–24.5) | 0.5% (0.0–1.6) | 0.0% (0.0–0.0) |
| 1999–2000 | Stage 2 | 1,916 | 52.0% (47.7–56.3) | 45.8% (41.5–50.0) | 0.4% (0.1–0.7) | 1.9% (1.3–2.5) |
| 1999–2000 | Stage 3 | 58 | 36.3% (19.5–53.2) | 48.7% (33.3–64.0) | 2.3% (0.0–5.1) | 12.7% (0.5–24.9) |
| 1999–2000 | Stage 4 | 284 | 39.3% (31.8–46.8) | 51.0% (42.1–59.9) | 2.1% (0.0–4.3) | 7.6% (4.8–10.5) |
| 2001–2002 | Overall | 2,740 | 54.4% (52.3–56.6) | 42.5% (40.2–44.8) | 0.5% (0.4–0.7) | 2.5% (2.0–3.0) |
| 2001–2002 | Stage 0 | 151 | 100.0% (100.0–100.0) | 0.0% (0.0–0.0) | 0.0% (0.0–0.0) | 0.0% (0.0–0.0) |
| 2001–2002 | Stage 1 | 189 | 77.8% (68.8–86.8) | 22.1% (13.2–31.0) | 0.0% (0.0–0.0) | 0.1% (0.0–0.4) |
| 2001–2002 | Stage 2 | 2,025 | 48.4% (46.2–50.7) | 49.4% (47.0–51.7) | 0.4% (0.3–0.6) | 1.8% (1.3–2.2) |
| 2001–2002 | Stage 3 | 75 | 47.2% (36.3–58.1) | 33.8% (19.9–47.6) | 0.0% (0.0–0.0) | 19.1% (8.1–30.0) |
| 2001–2002 | Stage 4 | 300 | 39.7% (31.6–47.9) | 46.9% (38.3–55.6) | 2.8% (1.1–4.4) | 10.6% (6.7–14.5) |
| 2003–2004 | Overall | 2,519 | 53.5% (51.2–55.8) | 42.7% (40.5–44.9) | 0.9% (0.6–1.2) | 2.9% (2.3–3.6) |
| 2003–2004 | Stage 0 | 110 | 100.0% (100.0–100.0) | 0.0% (0.0–0.0) | 0.0% (0.0–0.0) | 0.0% (0.0–0.0) |
| 2003–2004 | Stage 1 | 197 | 79.8% (74.1–85.5) | 20.0% (14.3–25.7) | 0.1% (0.0–0.3) | 0.2% (0.0–0.5) |
| 2003–2004 | Stage 2 | 1,802 | 47.1% (44.2–50.1) | 49.9% (47.2–52.7) | 0.7% (0.4–0.9) | 2.3% (1.5–3.0) |
| 2003–2004 | Stage 3 | 63 | 39.2% (30.3–48.1) | 40.8% (24.3–57.2) | 3.9% (0.0–9.1) | 16.1% (4.6–27.5) |
| 2003–2004 | Stage 4 | 347 | 44.5% (40.2–48.9) | 41.6% (36.8–46.4) | 3.1% (1.0–5.3) | 10.7% (8.2–13.3) |
| 2005–2006 | Overall | 2,569 | 49.7% (47.7–51.7) | 46.3% (44.3–48.3) | 0.7% (0.5–1.0) | 3.3% (2.5–4.1) |
| 2005–2006 | Stage 0 | 111 | 100.0% (100.0–100.0) | 0.0% (0.0–0.0) | 0.0% (0.0–0.0) | 0.0% (0.0–0.0) |
| 2005–2006 | Stage 1 | 185 | 78.6% (74.0–83.1) | 21.4% (16.9–26.0) | 0.0% (0.0–0.0) | 0.0% (0.0–0.0) |
| 2005–2006 | Stage 2 | 1,911 | 43.9% (41.5–46.2) | 53.1% (50.5–55.6) | 0.4% (0.1–0.6) | 2.7% (1.8–3.5) |
| 2005–2006 | Stage 3 | 65 | 51.2% (36.3–66.1) | 35.7% (19.5–52.0) | 1.3% (0.0–3.9) | 11.7% (3.2–20.2) |
| 2005–2006 | Stage 4 | 297 | 34.7% (29.2–40.2) | 48.3% (43.9–52.8) | 4.8% (2.6–6.9) | 12.2% (9.1–15.3) |
| 2007–2008 | Overall | 3,318 | 50.2% (47.2–53.2) | 45.1% (42.2–48.0) | 1.1% (0.9–1.3) | 3.6% (2.9–4.3) |
| 2007–2008 | Stage 0 | 113 | 100.0% (100.0–100.0) | 0.0% (0.0–0.0) | 0.0% (0.0–0.0) | 0.0% (0.0–0.0) |
| 2007–2008 | Stage 1 | 233 | 72.8% (64.9–80.8) | 26.9% (18.7–35.1) | 0.0% (0.0–0.0) | 0.2% (0.0–0.6) |
| 2007–2008 | Stage 2 | 2,520 | 45.4% (42.2–48.7) | 50.5% (47.4–53.7) | 0.7% (0.5–1.0) | 3.3% (2.3–4.3) |
| 2007–2008 | Stage 3 | 92 | 53.7% (38.9–68.5) | 36.7% (26.1–47.3) | 3.3% (0.0–7.4) | 6.4% (0.7–12.1) |
| 2007–2008 | Stage 4 | 360 | 37.7% (31.1–44.4) | 44.2% (36.4–52.0) | 6.1% (3.6–8.6) | 12.0% (7.6–16.3) |
| 2009–2010 | Overall | 3,427 | 49.7% (47.6–51.9) | 42.6% (40.2–45.0) | 1.6% (1.1–2.0) | 6.1% (5.3–6.9) |
| 2009–2010 | Stage 0 | 124 | 100.0% (100.0–100.0) | 0.0% (0.0–0.0) | 0.0% (0.0–0.0) | 0.0% (0.0–0.0) |
| 2009–2010 | Stage 1 | 257 | 81.0% (75.8–86.3) | 18.5% (13.4–23.6) | 0.0% (0.0–0.0) | 0.4% (0.0–1.1) |
| 2009–2010 | Stage 2 | 2,587 | 45.0% (42.6–47.4) | 49.2% (46.5–52.0) | 1.0% (0.7–1.4) | 4.8% (3.9–5.7) |
| 2009–2010 | Stage 3 | 93 | 28.2% (10.2–46.2) | 32.3% (21.1–43.6) | 7.0% (0.8–13.2) | 32.4% (21.0–43.9) |
| 2009–2010 | Stage 4 | 366 | 32.5% (26.6–38.3) | 37.6% (32.9–42.4) | 7.6% (4.1–11.2) | 22.3% (17.6–26.9) |
| 2011–2012 | Overall | 3,218 | 54.9% (52.4–57.5) | 38.0% (35.8–40.2) | 1.7% (1.2–2.2) | 5.4% (4.1–6.6) |
| 2011–2012 | Stage 0 | 297 | 100.0% (100.0–100.0) | 0.0% (0.0–0.0) | 0.0% (0.0–0.0) | 0.0% (0.0–0.0) |
| 2011–2012 | Stage 1 | 353 | 80.5% (75.6–85.5) | 19.5% (14.5–24.4) | 0.0% (0.0–0.0) | 0.0% (0.0–0.0) |
| 2011–2012 | Stage 2 | 2,213 | 47.1% (44.4–49.9) | 47.1% (44.4–49.7) | 1.0% (0.6–1.3) | 4.8% (3.5–6.2) |
| 2011–2012 | Stage 3 | 90 | 26.4% (16.1–36.7) | 33.2% (20.1–46.4) | 13.9% (0.9–26.8) | 26.5% (10.5–42.6) |
| 2011–2012 | Stage 4 | 265 | 28.3% (20.9–35.7) | 37.6% (31.2–43.9) | 11.3% (6.7–16.0) | 22.8% (14.6–30.9) |
| 2013–2014 | Overall | 3,648 | 51.7% (49.3–54.2) | 40.2% (37.8–42.7) | 1.4% (0.8–1.9) | 6.7% (5.8–7.5) |
| 2013–2014 | Stage 0 | 265 | 100.0% (100.0–100.0) | 0.0% (0.0–0.0) | 0.0% (0.0–0.0) | 0.0% (0.0–0.0) |
| 2013–2014 | Stage 1 | 427 | 78.6% (73.8–83.4) | 20.9% (16.2–25.5) | 0.0% (0.0–0.0) | 0.5% (0.0–1.4) |
| 2013–2014 | Stage 2 | 2,544 | 43.0% (40.7–45.2) | 50.0% (47.9–52.2) | 1.1% (0.7–1.6) | 5.9% (4.8–7.0) |
| 2013–2014 | Stage 3 | 86 | 24.4% (15.5–33.3) | 38.1% (30.1–46.1) | 11.8% (3.8–19.8) | 25.7% (10.0–41.4) |
| 2013–2014 | Stage 4 | 326 | 35.6% (29.9–41.4) | 32.2% (25.8–38.7) | 5.1% (1.9–8.3) | 27.1% (21.7–32.5) |
| 2015–2016 | Overall | 3,510 | 50.0% (46.1–53.8) | 42.8% (39.5–46.0) | 1.4% (1.1–1.8) | 5.9% (4.8–6.9) |
| 2015–2016 | Stage 0 | 245 | 100.0% (100.0–100.0) | 0.0% (0.0–0.0) | 0.0% (0.0–0.0) | 0.0% (0.0–0.0) |
| 2015–2016 | Stage 1 | 410 | 78.1% (73.2–83.0) | 21.2% (16.5–25.9) | 0.0% (0.0–0.0) | 0.7% (0.0–1.5) |
| 2015–2016 | Stage 2 | 2,430 | 40.5% (36.3–44.7) | 52.6% (48.7–56.5) | 1.5% (0.9–2.1) | 5.4% (4.2–6.6) |
| 2015–2016 | Stage 3 | 91 | 27.6% (14.1–41.0) | 51.0% (40.8–61.2) | 6.6% (0.0–13.2) | 14.9% (6.7–23.0) |
| 2015–2016 | Stage 4 | 334 | 33.5% (25.6–41.3) | 38.6% (30.4–46.9) | 3.8% (1.8–5.9) | 24.1% (16.8–31.3) |
| 2017–2018 | Overall | 3,822 | 45.5% (41.7–49.4) | 44.5% (41.2–47.8) | 1.8% (1.3–2.3) | 8.2% (7.1–9.2) |
| 2017–2018 | Stage 0 | 230 | 99.8% (99.5–100.0) | 0.0% (0.0–0.0) | 0.2% (0.0–0.5) | 0.0% (0.0–0.0) |
| 2017–2018 | Stage 1 | 389 | 73.8% (67.6–80.1) | 25.7% (19.5–31.8) | 0.2% (0.0–0.5) | 0.3% (0.0–0.6) |
| 2017–2018 | Stage 2 | 2,614 | 37.1% (33.4–40.9) | 53.8% (50.6–57.0) | 1.4% (1.0–1.8) | 7.7% (6.4–8.9) |
| 2017–2018 | Stage 3 | 107 | 21.6% (8.2–35.1) | 53.1% (42.1–64.1) | 7.0% (2.6–11.4) | 18.2% (13.1–23.3) |
| 2017–2018 | Stage 4 | 482 | 26.1% (18.8–33.5) | 39.9% (32.0–47.8) | 7.2% (3.8–10.6) | 26.8% (21.9–31.7) |

***Note.*** *The table is a wide-format presentation of all 240 cycle–stage–phenotype estimates used in Supplementary Figure S1. Estimates are unadjusted descriptive survey-weighted proportions; no formal time-trend test is reported.*

**Supplementary Table S6. Kaplan–Meier support, landmark risk sets, survival estimates, and design-based log-rank tests**

**Panel A. Supported phenotype curves and landmark survival**

| **CKM stage** | **Phenotype** | **N at 0 years** | **Deaths** | **5-year survival (n at risk)** | **10-year survival (n at risk)** | **15-year survival (n at risk)** |
| --- | --- | --- | --- | --- | --- | --- |
| Stage 1 | A | 2,163 | 55 | 99.6% (n=1561) | 98.5% (n=821) | 95.9% (n=407) |
| Stage 1 | B | 628 | 19 | 98.7% (n=429) | 97.3% (n=239) | 96.3% (n=112) |
| Stage 2 | A | 9,981 | 1,188 | 97.5% (n=7702) | 93.2% (n=4772) | 86.8% (n=2208) |
| Stage 2 | B | 10,963 | 963 | 98.3% (n=8292) | 94.8% (n=5239) | 89.8% (n=2315) |
| Stage 2 | C | 348 | 123 | 83.0% (n=196) | 55.1% (n=70) | 36.2% (n=19) |
| Stage 2 | D | 1,270 | 267 | 89.9% (n=767) | 77.7% (n=337) | 58.8% (n=87) |
| Stage 3 | A | 267 | 100 | 84.0% (n=182) | 66.6% (n=90) | 49.7% (n=31) |
| Stage 3 | B | 327 | 101 | 90.6% (n=210) | 73.2% (n=110) | 49.3% (n=36) |
| Stage 3 | C | 51 | 25 | 59.9% (n=24) | Truncated (n=8) | Truncated (n=2) |
| Stage 3 | D | 175 | 78 | 73.6% (n=91) | 43.6% (n=26) | Truncated (n=5) |
| Stage 4 | A | 1,156 | 474 | 87.5% (n=763) | 67.1% (n=395) | 50.6% (n=159) |
| Stage 4 | B | 1,322 | 457 | 89.3% (n=889) | 73.4% (n=532) | 57.5% (n=201) |
| Stage 4 | C | 219 | 110 | 68.3% (n=111) | 41.7% (n=36) | Truncated (n=9) |
| Stage 4 | D | 664 | 268 | 80.1% (n=346) | 50.8% (n=122) | 21.6% (n=17) |

**Panel B. Design-based log-rank tests**

| **CKM stage** | **Groups** | **N** | **Deaths** | **Statistic** | **df** | **P value** | **Status** |
| --- | --- | --- | --- | --- | --- | --- | --- |
| Stage 0 | — | 1,768 | 50 | — | — | — | Not Estimable |
| Stage 1 | A;B | 2,791 | 74 | 0.154 | 1 | 0.695 | Estimable |
| Stage 2 | A;B;C;D | 22,562 | 2,541 | 142.707 | 3 | <0.001 | Estimable |
| Stage 3 | A;B;C;D | 820 | 304 | 31.438 | 3 | <0.001 | Estimable |
| Stage 4 | A;B;C;D | 3,361 | 1,309 | 81.247 | 3 | <0.001 | Estimable |

***Note.*** *Unadjusted survey-weighted Kaplan–Meier estimates used the 20-year MEC weights and cycle-nested strata and primary sampling units. N at risk is unweighted. Curves required at least 30 participants and five deaths; values were not displayed once fewer than 10 participants remained at risk. Stage 0 lacked comparative phenotype support. Stage 3 phenotype C had eight participants at risk at 10 years and is therefore not reported at that landmark.*

**Supplementary Table S7. Weighted Aalen–Johansen cardiovascular cumulative-incidence support and landmark risk sets**

| **CKM stage** | **Phenotype** | **N at 0 years** | **CV deaths** | **Competing deaths** | **5-year CIF (n at risk)** | **10-year CIF (n at risk)** | **15-year CIF (n at risk)** |
| --- | --- | --- | --- | --- | --- | --- | --- |
| Stage 2 | A | 9,981 | 343 | 845 | 0.6% (n=7702) | 1.8% (n=4772) | 3.5% (n=2208) |
| Stage 2 | B | 10,963 | 274 | 689 | 0.4% (n=8292) | 1.3% (n=5239) | 2.8% (n=2315) |
| Stage 2 | C | 348 | 32 | 91 | 5.5% (n=196) | 9.9% (n=70) | 16.2% (n=19) |
| Stage 2 | D | 1,270 | 99 | 168 | 3.8% (n=767) | 8.5% (n=337) | 18.6% (n=87) |
| Stage 3 | A | 267 | 31 | 69 | 6.9% (n=182) | 13.7% (n=90) | 15.7% (n=31) |
| Stage 3 | B | 327 | 27 | 74 | 2.2% (n=210) | 7.0% (n=110) | 11.1% (n=36) |
| Stage 3 | C | 51 | 7 | 18 | 5.5% (n=24) | Truncated (n=8) | Truncated (n=2) |
| Stage 3 | D | 175 | 25 | 53 | 4.9% (n=91) | 16.4% (n=26) | Truncated (n=5) |
| Stage 4 | A | 1,156 | 201 | 273 | 5.3% (n=763) | 14.4% (n=395) | 19.9% (n=159) |
| Stage 4 | B | 1,322 | 184 | 273 | 4.6% (n=889) | 10.0% (n=532) | 17.1% (n=201) |
| Stage 4 | C | 219 | 52 | 58 | 16.3% (n=111) | 28.2% (n=36) | Truncated (n=9) |
| Stage 4 | D | 664 | 115 | 153 | 8.5% (n=346) | 17.1% (n=122) | 31.2% (n=17) |

***Note.*** *Cumulative incidence functions are descriptive 20-year MEC-weighted Aalen–Johansen point estimates, with non-cardiovascular death treated as a competing event. N at risk is unweighted. Stage 1 was unsupported. Tails were truncated below 10 participants at risk. No Gray test, Fine–Gray model, subdistribution hazard ratio, inferential cumulative-incidence P value, or design-based cumulative-incidence confidence interval is reported.*

**Supplementary Table S8. Joint-phenotype interaction tests for CKM stage and cardiometabolic risk-factor burden**

| **Outcome** | **Interaction** | **Wald statistic** | **df** | **P value** | **N** | **Events** | **Imputations** | **Status or reason** |
| --- | --- | --- | --- | --- | --- | --- | --- | --- |
| All-cause mortality | Phenotype × CKM stage (global Wald) | — | — | — | 31,317 | 4,284 | 0 | Structurally non-estimable |
| All-cause mortality | Phenotype × CKM stage (support set: stages 2–4; global Wald) | 10.284 | 6 | 0.113 | 26,743 | 4,154 | 20 | — |
| Cardiovascular mortality | Phenotype × CKM stage (global Wald) | — | — | — | 31,317 | 1,417 | 0 | Structurally non-estimable |
| Cardiovascular mortality | Phenotype × CKM stage (support set: stages 2–4; global Wald) | 10.058 | 6 | 0.122 | 26,743 | 1,390 | 20 | — |
| All-cause mortality | Phenotype × CMRF group (global Wald) | 8.760 | 6 | 0.188 | 27,027 | 3,570 | 20 | — |
| Cardiovascular mortality | Phenotype × CMRF group (global Wald) | 10.998 | 6 | 0.088 | 27,027 | 1,169 | 20 | — |

***Note.*** *The complete phenotype-by-stage interaction across stages 0–4 was attempted but was structurally non-estimable because early stages lacked all four phenotype cells. The stages 2–4 support-set test is reported as a separate estimable analysis, not as a substitute for the unsupported full interaction. CMRF, cardiometabolic risk factor.*

**Supplementary Table S9. Restricted-cubic-spline support, modelling range, reference, and knot audit**

| **Outcome** | **CKM stage** | **Status** | **N (events)** | **Grid points** | **NFS modelling range** | **Reference NFS** | **Knots** | **Reason** |
| --- | --- | --- | --- | --- | --- | --- | --- | --- |
| All-cause mortality | Stage 0 | Not estimable | 1,768 (50) | 0 | — | — | — | Spline suppressed for inadequate support across NFS-low/high strata. Sparse cell: Observed Low n=1767/events=50; High n=1/events=0 |
| All-cause mortality | Stage 1 | Not estimable | 2,806 (80) | 0 | — | — | — | Spline suppressed for inadequate support across NFS-low/high strata. Sparse cell: Observed Low n=2791/events=74; High n=15/events=6 |
| All-cause mortality | Stage 2 | Estimable | 22,562 (2,541) | 81 | -4.676 to 1.537 | -1.599 | -4.091; -2.245; -0.953; 0.963 | — |
| All-cause mortality | Stage 3 | Estimable | 820 (304) | 81 | -3.885 to 2.591 | -0.226 | -3.147; -0.829; 0.341; 2.003 | — |
| All-cause mortality | Stage 4 | Estimable | 3,361 (1,309) | 81 | -3.604 to 2.539 | -0.217 | -2.855; -0.823; 0.324; 1.949 | — |
| Cardiovascular mortality | Stage 0 | Not estimable | 1,768 (10) | 0 | — | — | — | Spline suppressed for inadequate support across NFS-low/high strata. Sparse cell: Observed Low n=1767/events=10; High n=1/events=0 |
| Cardiovascular mortality | Stage 1 | Not estimable | 2,806 (17) | 0 | — | — | — | Spline suppressed for inadequate support across NFS-low/high strata. Sparse cell: Observed Low n=2791/events=15; High n=15/events=2 |
| Cardiovascular mortality | Stage 2 | Estimable | 22,562 (748) | 81 | -4.676 to 1.537 | -1.599 | -4.091; -2.245; -0.953; 0.963 | — |
| Cardiovascular mortality | Stage 3 | Estimable | 820 (90) | 81 | -3.885 to 2.591 | -0.226 | -3.147; -0.829; 0.341; 2.003 | — |
| Cardiovascular mortality | Stage 4 | Estimable | 3,361 (552) | 81 | -3.604 to 2.539 | -0.217 | -2.855; -0.823; 0.324; 1.949 | — |

***Note.*** *Continuous NFS was modelled only in stages 2–4, using stage-specific knots at the 5th, 35th, 65th, and 95th percentiles and the within-stage median as reference. Entire stage 0–1 panels were suppressed for inadequate NFS-low/high support. No formal non-linearity P value was generated.*

**Supplementary Table S10. Apparent weighted Harrell C and incremental in-sample discrimination**

| **Analysis population** | **Model** | **Harrell C (95% CI)** | **Change from base** | **N** | **Events** |
| --- | --- | --- | --- | --- | --- |
| Overall | base | 0.8563 (0.8467–0.8659) | +0.0000 | 31,317 | 4,284 |
| Overall | +MASLD | 0.8566 (0.8471–0.8662) | +0.0004 | 31,317 | 4,284 |
| Overall | +NFS | 0.8580 (0.8484–0.8677) | +0.0018 | 31,317 | 4,284 |
| Overall | +joint | 0.8583 (0.8487–0.8679) | +0.0020 | 31,317 | 4,284 |
| Stage 0 | base | 0.8042 (0.6464–0.9621) | +0.0000 | 1,768 | 50 |
| Stage 0 | +MASLD | 0.8042 (0.6464–0.9621) | +0.0000 | 1,768 | 50 |
| Stage 0 | +NFS | 0.8071 (0.6524–0.9617) | +0.0028 | 1,768 | 50 |
| Stage 0 | +joint | 0.8043 (0.6465–0.9621) | +0.0000 | 1,768 | 50 |
| Stage 1 | base | 0.8399 (0.7696–0.9102) | +0.0000 | 2,806 | 80 |
| Stage 1 | +MASLD | 0.8398 (0.7689–0.9107) | -0.0001 | 2,806 | 80 |
| Stage 1 | +NFS | 0.8383 (0.7676–0.9091) | -0.0016 | 2,806 | 80 |
| Stage 1 | +joint | 0.8431 (0.7718–0.9143) | +0.0031 | 2,806 | 80 |
| Stage 2 | base | 0.8304 (0.8171–0.8436) | +0.0000 | 22,562 | 2,541 |
| Stage 2 | +MASLD | 0.8304 (0.8171–0.8436) | +0.0000 | 22,562 | 2,541 |
| Stage 2 | +NFS | 0.8313 (0.8181–0.8446) | +0.0010 | 22,562 | 2,541 |
| Stage 2 | +joint | 0.8319 (0.8187–0.8451) | +0.0015 | 22,562 | 2,541 |
| Stage 3 | base | 0.7439 (0.7021–0.7858) | +0.0000 | 820 | 304 |
| Stage 3 | +MASLD | 0.7445 (0.7022–0.7868) | +0.0005 | 820 | 304 |
| Stage 3 | +NFS | 0.7506 (0.7110–0.7902) | +0.0067 | 820 | 304 |
| Stage 3 | +joint | 0.7589 (0.7198–0.7980) | +0.0150 | 820 | 304 |
| Stage 4 | base | 0.7592 (0.7397–0.7787) | +0.0000 | 3,361 | 1,309 |
| Stage 4 | +MASLD | 0.7596 (0.7402–0.7789) | +0.0004 | 3,361 | 1,309 |
| Stage 4 | +NFS | 0.7616 (0.7422–0.7811) | +0.0024 | 3,361 | 1,309 |
| Stage 4 | +joint | 0.7606 (0.7416–0.7797) | +0.0014 | 3,361 | 1,309 |

***Note.*** *These are apparent in-sample estimates obtained with mean-one-rescaled survey weights and cluster-robust standard errors, averaged across imputations. No external validation, calibration, time-dependent AUC, Brier score, NRI, IDI, or decision-curve analysis was performed; changes must not be interpreted as demonstrated clinical utility.*

**Supplementary Table S11. Exploratory D-versus-A subgroup effects, interaction tests, and availability**

**Panel A. Subgroup-specific effects**

| **Outcome** | **Subgroup** | **Level** | **N (events)** | **A n/events** | **D n/events** | **HR (95% CI)** | **P value** | **P interaction** | **Available/missing N** |
| --- | --- | --- | --- | --- | --- | --- | --- | --- | --- |
| All-cause mortality | Age | <60 years | 7,314 (293) | 6,853/247 | 461/46 | 5.36 (3.50–8.21) | <0.001 | <0.001 | 26,743/0 missing |
| All-cause mortality | Age | ≥60 years | 6,199 (2,082) | 4,551/1,515 | 1,648/567 | 1.36 (1.21–1.53) | <0.001 | <0.001 | 26,743/0 missing |
| All-cause mortality | Sex | Female | 7,616 (1,233) | 6,495/942 | 1,121/291 | 1.55 (1.32–1.83) | <0.001 | 0.786 | 26,743/0 missing |
| All-cause mortality | Sex | Male | 5,897 (1,142) | 4,909/820 | 988/322 | 1.50 (1.25–1.80) | <0.001 | 0.786 | 26,743/0 missing |
| All-cause mortality | Race/ethnicity | Non-Hispanic White | 6,083 (1,508) | 5,106/1,140 | 977/368 | 1.58 (1.37–1.81) | <0.001 | 0.584 | 26,743/0 missing |
| All-cause mortality | Race/ethnicity | Non-White | 7,430 (867) | 6,298/622 | 1,132/245 | 1.47 (1.18–1.82) | <0.001 | 0.584 | 26,743/0 missing |
| All-cause mortality | Diabetes status | No diabetes | 11,299 (1,801) | 10,271/1,514 | 1,028/287 | 1.42 (1.20–1.67) | <0.001 | 0.726 | 26,739/4 missing |
| All-cause mortality | Diabetes status | Diabetes | 2,213 (573) | 1,132/247 | 1,081/326 | 1.35 (1.07–1.69) | 0.011 | 0.726 | 26,739/4 missing |
| All-cause mortality | Body mass index | BMI <30 kg/m² | 10,980 (1,837) | 10,684/1,703 | 296/134 | 1.76 (1.42–2.17) | <0.001 | 0.481 | 26,743/0 missing |
| All-cause mortality | Body mass index | BMI ≥30 kg/m² | 2,533 (538) | 720/59 | 1,813/479 | 1.53 (1.11–2.11) | 0.009 | 0.481 | 26,743/0 missing |
| All-cause mortality | Hypertension | No hypertension | 4,982 (375) | 4,714/305 | 268/70 | 2.03 (1.38–2.97) | <0.001 | 0.083 | 26,414/329 missing |
| All-cause mortality | Hypertension | Hypertension | 8,362 (1,972) | 6,546/1,439 | 1,816/533 | 1.43 (1.26–1.62) | <0.001 | 0.083 | 26,414/329 missing |
| All-cause mortality | Chronic kidney disease | No CKD | 10,478 (1,262) | 9,266/1,029 | 1,212/233 | 1.45 (1.21–1.73) | <0.001 | 0.839 | 26,721/22 missing |
| All-cause mortality | Chronic kidney disease | CKD | 3,020 (1,106) | 2,130/727 | 890/379 | 1.49 (1.26–1.74) | <0.001 | 0.839 | 26,721/22 missing |
| All-cause mortality | Smoking status | Never smoker | 7,814 (1,120) | 6,692/846 | 1,122/274 | 1.42 (1.20–1.69) | <0.001 | 0.788 | 26,743/0 missing |
| All-cause mortality | Smoking status | Ever smoker | 5,699 (1,255) | 4,712/916 | 987/339 | 1.47 (1.25–1.73) | <0.001 | 0.788 | 26,743/0 missing |
| All-cause mortality | Lipid-lowering medication use | No lipid-lowering medication | 7,043 (1,147) | 6,083/858 | 960/289 | 1.50 (1.28–1.77) | <0.001 | 0.535 | 19,509/7,234 missing |
| All-cause mortality | Lipid-lowering medication use | Lipid-lowering medication | 2,906 (681) | 2,037/451 | 869/230 | 1.64 (1.33–2.02) | <0.001 | 0.535 | 19,509/7,234 missing |
| All-cause mortality | Antihypertensive medication use | No antihypertensive medication | 8,719 (1,086) | 8,040/898 | 679/188 | 1.74 (1.40–2.15) | <0.001 | 0.157 | 25,329/1,414 missing |
| All-cause mortality | Antihypertensive medication use | Antihypertensive medication | 4,203 (1,192) | 2,828/780 | 1,375/412 | 1.42 (1.22–1.66) | <0.001 | 0.157 | 25,329/1,414 missing |
| All-cause mortality | Glucose-lowering medication use | No glucose-lowering medication | 13,000 (2,225) | 11,188/1,712 | 1,812/513 | 1.48 (1.30–1.69) | <0.001 | 0.132 | 26,740/3 missing |
| All-cause mortality | Glucose-lowering medication use | Glucose-lowering medication | 512 (149) | 215/49 | 297/100 | 1.07 (0.71–1.61) | 0.746 | 0.132 | 26,740/3 missing |
| Cardiovascular mortality | Age | <60 years | 7,314 (75) | 6,853/62 | 461/13 | 5.33 (2.67–10.65) | <0.001 | <0.001 | 26,743/0 missing |
| Cardiovascular mortality | Age | ≥60 years | 6,199 (739) | 4,551/513 | 1,648/226 | 1.48 (1.22–1.81) | <0.001 | <0.001 | 26,743/0 missing |
| Cardiovascular mortality | Sex | Female | 7,616 (394) | 6,495/284 | 1,121/110 | 1.64 (1.25–2.14) | <0.001 | 0.996 | 26,743/0 missing |
| Cardiovascular mortality | Sex | Male | 5,897 (420) | 4,909/291 | 988/129 | 1.64 (1.25–2.16) | <0.001 | 0.996 | 26,743/0 missing |
| Cardiovascular mortality | Race/ethnicity | Non-Hispanic White | 6,083 (533) | 5,106/384 | 977/149 | 1.68 (1.34–2.11) | <0.001 | 0.741 | 26,743/0 missing |
| Cardiovascular mortality | Race/ethnicity | Non-White | 7,430 (281) | 6,298/191 | 1,132/90 | 1.58 (1.14–2.17) | 0.005 | 0.741 | 26,743/0 missing |
| Cardiovascular mortality | Diabetes status | No diabetes | 11,299 (603) | 10,271/489 | 1,028/114 | 1.60 (1.23–2.08) | <0.001 | 0.315 | 26,739/4 missing |
| Cardiovascular mortality | Diabetes status | Diabetes | 2,213 (211) | 1,132/86 | 1,081/125 | 1.27 (0.87–1.84) | 0.217 | 0.315 | 26,739/4 missing |
| Cardiovascular mortality | Body mass index | BMI <30 kg/m² | 10,980 (617) | 10,684/557 | 296/60 | 2.26 (1.59–3.21) | <0.001 | 0.294 | 26,743/0 missing |
| Cardiovascular mortality | Body mass index | BMI ≥30 kg/m² | 2,533 (197) | 720/18 | 1,813/179 | 1.51 (0.82–2.79) | 0.190 | 0.294 | 26,743/0 missing |
| Cardiovascular mortality | Hypertension | No hypertension | 4,982 (97) | 4,714/72 | 268/25 | 2.34 (1.29–4.23) | 0.005 | 0.176 | 26,414/329 missing |
| Cardiovascular mortality | Hypertension | Hypertension | 8,362 (700) | 6,546/491 | 1,816/209 | 1.49 (1.21–1.85) | <0.001 | 0.176 | 26,414/329 missing |
| Cardiovascular mortality | Chronic kidney disease | No CKD | 10,478 (393) | 9,266/309 | 1,212/84 | 1.73 (1.29–2.32) | <0.001 | 0.360 | 26,721/22 missing |
| Cardiovascular mortality | Chronic kidney disease | CKD | 3,020 (420) | 2,130/265 | 890/155 | 1.44 (1.12–1.86) | 0.005 | 0.360 | 26,721/22 missing |
| Cardiovascular mortality | Smoking status | Never smoker | 7,814 (419) | 6,692/300 | 1,122/119 | 1.56 (1.21–2.01) | <0.001 | 0.941 | 26,743/0 missing |
| Cardiovascular mortality | Smoking status | Ever smoker | 5,699 (395) | 4,712/275 | 987/120 | 1.58 (1.19–2.10) | 0.002 | 0.941 | 26,743/0 missing |
| Cardiovascular mortality | Lipid-lowering medication use | No lipid-lowering medication | 7,043 (372) | 6,083/259 | 960/113 | 1.88 (1.41–2.51) | <0.001 | 0.175 | 19,509/7,234 missing |
| Cardiovascular mortality | Lipid-lowering medication use | Lipid-lowering medication | 2,906 (247) | 2,037/161 | 869/86 | 1.34 (0.92–1.96) | 0.124 | 0.175 | 19,509/7,234 missing |
| Cardiovascular mortality | Antihypertensive medication use | No antihypertensive medication | 8,719 (348) | 8,040/275 | 679/73 | 2.03 (1.42–2.90) | <0.001 | 0.125 | 25,329/1,414 missing |
| Cardiovascular mortality | Antihypertensive medication use | Antihypertensive medication | 4,203 (436) | 2,828/275 | 1,375/161 | 1.43 (1.11–1.84) | 0.005 | 0.125 | 25,329/1,414 missing |
| Cardiovascular mortality | Glucose-lowering medication use | No glucose-lowering medication | 13,000 (762) | 11,188/560 | 1,812/202 | 1.57 (1.27–1.93) | <0.001 | 0.996 | 26,740/3 missing |
| Cardiovascular mortality | Glucose-lowering medication use | Glucose-lowering medication | 512 (52) | 215/15 | 297/37 | 1.57 (0.70–3.54) | 0.278 | 0.996 | 26,740/3 missing |

**Panel B. Nominal interaction tests**

| **Outcome** | **Subgroup** | **Comparison** | **N (events)** | **Wald statistic** | **P interaction** | **Status** |
| --- | --- | --- | --- | --- | --- | --- |
| All-cause mortality | Age | <60 years vs ≥60 years | 13,513 (2,375) | 38.383 | <0.001 | Estimable |
| All-cause mortality | Sex | Female vs Male | 13,513 (2,375) | 0.074 | 0.786 | Estimable |
| All-cause mortality | Race/ethnicity | Non-Hispanic White vs Non-White | 13,513 (2,375) | 0.301 | 0.584 | Estimable |
| All-cause mortality | Diabetes status | No diabetes vs Diabetes | 13,512 (2,374) | 0.123 | 0.726 | Estimable |
| All-cause mortality | Body mass index | BMI <30 kg/m² vs BMI ≥30 kg/m² | 13,513 (2,375) | 0.496 | 0.481 | Estimable |
| All-cause mortality | Hypertension | No hypertension vs Hypertension | 13,344 (2,347) | 3.005 | 0.083 | Estimable |
| All-cause mortality | Chronic kidney disease | No CKD vs CKD | 13,498 (2,368) | 0.041 | 0.839 | Estimable |
| All-cause mortality | Smoking status | Never smoker vs Ever smoker | 13,513 (2,375) | 0.072 | 0.788 | Estimable |
| All-cause mortality | Lipid-lowering medication use | No lipid-lowering medication vs Lipid-lowering medication | 9,949 (1,828) | 0.385 | 0.535 | Estimable |
| All-cause mortality | Antihypertensive medication use | No antihypertensive medication vs Antihypertensive medication | 12,922 (2,278) | 2.008 | 0.157 | Estimable |
| All-cause mortality | Glucose-lowering medication use | No glucose-lowering medication vs Glucose-lowering medication | 13,512 (2,374) | 2.263 | 0.132 | Estimable |
| Cardiovascular mortality | Age | <60 years vs ≥60 years | 13,513 (814) | 11.768 | <0.001 | Estimable |
| Cardiovascular mortality | Sex | Female vs Male | 13,513 (814) | 0.000 | 0.996 | Estimable |
| Cardiovascular mortality | Race/ethnicity | Non-Hispanic White vs Non-White | 13,513 (814) | 0.109 | 0.741 | Estimable |
| Cardiovascular mortality | Diabetes status | No diabetes vs Diabetes | 13,512 (814) | 1.009 | 0.315 | Estimable |
| Cardiovascular mortality | Body mass index | BMI <30 kg/m² vs BMI ≥30 kg/m² | 13,513 (814) | 1.101 | 0.294 | Estimable |
| Cardiovascular mortality | Hypertension | No hypertension vs Hypertension | 13,344 (797) | 1.833 | 0.176 | Estimable |
| Cardiovascular mortality | Chronic kidney disease | No CKD vs CKD | 13,498 (813) | 0.837 | 0.360 | Estimable |
| Cardiovascular mortality | Smoking status | Never smoker vs Ever smoker | 13,513 (814) | 0.005 | 0.941 | Estimable |
| Cardiovascular mortality | Lipid-lowering medication use | No lipid-lowering medication vs Lipid-lowering medication | 9,949 (619) | 1.841 | 0.175 | Estimable |
| Cardiovascular mortality | Antihypertensive medication use | No antihypertensive medication vs Antihypertensive medication | 12,922 (784) | 2.353 | 0.125 | Estimable |
| Cardiovascular mortality | Glucose-lowering medication use | No glucose-lowering medication vs Glucose-lowering medication | 13,512 (814) | 0.000 | 0.996 | Estimable |

**Panel C. Medication availability and alcohol estimability**

| **Dimension** | **Available N** | **Missing n** | **Status** | **Reason** |
| --- | --- | --- | --- | --- |
| Lipid-lowering medication use | 19,509 | 7,234 | Available-case analysis | — |
| Antihypertensive medication use | 25,329 | 1,414 | Available-case analysis | — |
| Glucose-lowering medication use | 26,740 | 3 | Available-case analysis | — |
| Heavy alcohol (MetALD/ALD) | 26,743 | 0 | By-design not estimable | Alcohol status was available in the stages 2–4 support set, but MetALD, ALD, and unknown intake had been excluded before the primary cohort was defined; observed heavy-alcohol n=0. |

***Note.*** *D was compared with A within the CKM stages 2–4 support set. Survey-weighted models included categorical CKM stage and the primary adjustment set, with the stratifying variable removed where applicable. Interaction tests were nominal and not multiplicity-adjusted. Medication variables retained their source labels and were analysed using observed-status available cases.*

**Supplementary Table S12. Cardiometabolic risk-factor burden and mortality analyses**

| **Outcome** | **Burden specification** | **Contrast** | **HR (95% CI)** | **P value** | **N** | **Events** | **Status or reason** |
| --- | --- | --- | --- | --- | --- | --- | --- |
| All-cause mortality | Continuous 0–5 | Per 1 additional CMRF | 1.05 (1.02–1.09) | 0.004 | 27,027 | 3,570 | — |
| All-cause mortality | CMRF 0-1 | B vs A | 0.87 (0.51–1.48) | 0.598 | 6,408 | 360 | — |
| All-cause mortality | CMRF 0-1 | D vs C | — | — | 6,408 | 360 | Sparse cell: Observed C n=19/events=8; D n=20/events=8 |
| All-cause mortality | CMRF 2-3 | B vs A | 1.01 (0.88–1.15) | 0.910 | 12,238 | 1,559 | — |
| All-cause mortality | CMRF 2-3 | D vs C | 1.31 (0.95–1.79) | 0.097 | 12,238 | 1,559 | — |
| All-cause mortality | CMRF 4-5 | B vs A | 1.02 (0.86–1.22) | 0.797 | 8,381 | 1,651 | — |
| All-cause mortality | CMRF 4-5 | D vs C | 0.98 (0.78–1.23) | 0.846 | 8,381 | 1,651 | — |
| Cardiovascular mortality | Continuous 0–5 | Per 1 additional CMRF | 1.15 (1.07–1.24) | <0.001 | 27,027 | 1,169 | — |
| Cardiovascular mortality | CMRF 0-1 | B vs A | 0.38 (0.12–1.24) | 0.110 | 6,408 | 81 | — |
| Cardiovascular mortality | CMRF 0-1 | D vs C | — | — | 6,408 | 81 | Sparse cell: Observed C n=19/events=1; D n=20/events=1 |
| Cardiovascular mortality | CMRF 2-3 | B vs A | 0.96 (0.76–1.21) | 0.705 | 12,238 | 505 | — |
| Cardiovascular mortality | CMRF 2-3 | D vs C | 1.78 (1.08–2.94) | 0.024 | 12,238 | 505 | — |
| Cardiovascular mortality | CMRF 4-5 | B vs A | 1.02 (0.77–1.35) | 0.888 | 8,381 | 583 | — |
| Cardiovascular mortality | CMRF 4-5 | D vs C | 0.94 (0.67–1.34) | 0.745 | 8,381 | 583 | — |

***Note.*** *CMRF count was available for 27,027 participants; 4,290 had missing CMRF count and this field was not imputed. Models used the prespecified adjustment set and Rubin-pooled survey-weighted covariance. CMRF, cardiometabolic risk factor.*

**Supplementary Table S13. Prespecified mortality sensitivity analyses**

| **Analysis** | **Outcome** | **CKM stage** | **Contrast** | **HR (95% CI)** | **P value** | **N (events)** | **Status or reason** |
| --- | --- | --- | --- | --- | --- | --- | --- |
| 2-year landmark | All-cause mortality | Stage 0 | Model not estimable | — | — | — | Sparse cell: Observed C n=0/events=0; D n=0/events=0 |
| 2-year landmark | All-cause mortality | Stage 1 | B vs A | 0.68 (0.31–1.47) | 0.328 | 2,609 (68) | — |
| 2-year landmark | All-cause mortality | Stage 1 | D vs C | — | — | 2,609 (68) | Sparse cell: Observed C n=2/events=2; D n=9/events=2 |
| 2-year landmark | All-cause mortality | Stage 2 | B vs A | 0.97 (0.86–1.08) | 0.569 | 21,001 (2,313) | — |
| 2-year landmark | All-cause mortality | Stage 2 | D vs C | 1.10 (0.84–1.44) | 0.505 | 21,001 (2,313) | — |
| 2-year landmark | All-cause mortality | Stage 3 | B vs A | 0.98 (0.72–1.35) | 0.921 | 719 (256) | — |
| 2-year landmark | All-cause mortality | Stage 3 | D vs C | 0.88 (0.43–1.82) | 0.739 | 719 (256) | — |
| 2-year landmark | All-cause mortality | Stage 4 | B vs A | 1.18 (0.97–1.44) | 0.090 | 2,931 (1,124) | — |
| 2-year landmark | All-cause mortality | Stage 4 | D vs C | 1.30 (0.97–1.74) | 0.081 | 2,931 (1,124) | — |
| 2-year landmark | Cardiovascular mortality | Stage 0 | Model not estimable | — | — | — | Sparse cell: Observed C n=0/events=0; D n=0/events=0 |
| 2-year landmark | Cardiovascular mortality | Stage 1 | B vs A | — | — | 2,609 (17) | Sparse cell: Observed A n=2027/events=12; B n=571/events=3 |
| 2-year landmark | Cardiovascular mortality | Stage 1 | D vs C | — | — | 2,609 (17) | Sparse cell: Observed C n=2/events=1; D n=9/events=1 |
| 2-year landmark | Cardiovascular mortality | Stage 2 | B vs A | 0.99 (0.81–1.22) | 0.957 | 21,001 (695) | — |
| 2-year landmark | Cardiovascular mortality | Stage 2 | D vs C | 1.56 (0.97–2.51) | 0.065 | 21,001 (695) | — |
| 2-year landmark | Cardiovascular mortality | Stage 3 | B vs A | 0.65 (0.34–1.25) | 0.199 | 719 (76) | — |
| 2-year landmark | Cardiovascular mortality | Stage 3 | D vs C | 1.21 (0.40–3.63) | 0.736 | 719 (76) | — |
| 2-year landmark | Cardiovascular mortality | Stage 4 | B vs A | 1.14 (0.82–1.58) | 0.449 | 2,931 (473) | — |
| 2-year landmark | Cardiovascular mortality | Stage 4 | D vs C | 1.03 (0.72–1.47) | 0.866 | 2,931 (473) | — |
| Complete-case | All-cause mortality | Stage 0 | Model not estimable | — | — | — | Sparse cell: Observed C n=1/events=0; D n=0/events=0 |
| Complete-case | All-cause mortality | Stage 1 | B vs A | 1.10 (0.54–2.24) | 0.800 | 2,625 (72) | — |
| Complete-case | All-cause mortality | Stage 1 | D vs C | — | — | 2,625 (72) | Sparse cell: Observed C n=2/events=1; D n=11/events=3 |
| Complete-case | All-cause mortality | Stage 2 | B vs A | 0.97 (0.87–1.09) | 0.658 | 20,642 (2,300) | — |
| Complete-case | All-cause mortality | Stage 2 | D vs C | 1.06 (0.83–1.35) | 0.648 | 20,642 (2,300) | — |
| Complete-case | All-cause mortality | Stage 3 | B vs A | 1.05 (0.76–1.44) | 0.770 | 738 (278) | — |
| Complete-case | All-cause mortality | Stage 3 | D vs C | 1.13 (0.54–2.33) | 0.750 | 738 (278) | — |
| Complete-case | All-cause mortality | Stage 4 | B vs A | 1.14 (0.93–1.40) | 0.204 | 3,087 (1,202) | — |
| Complete-case | All-cause mortality | Stage 4 | D vs C | 1.13 (0.85–1.50) | 0.392 | 3,087 (1,202) | — |
| Complete-case | Cardiovascular mortality | Stage 0 | Model not estimable | — | — | — | Sparse cell: Observed C n=1/events=0; D n=0/events=0 |
| Complete-case | Cardiovascular mortality | Stage 1 | Model not estimable | — | — | — | Sparse cell: Observed C n=2/events=0; D n=11/events=1 |
| Complete-case | Cardiovascular mortality | Stage 2 | B vs A | 1.01 (0.81–1.24) | 0.953 | 20,642 (684) | — |
| Complete-case | Cardiovascular mortality | Stage 2 | D vs C | 1.69 (1.05–2.73) | 0.031 | 20,642 (684) | — |
| Complete-case | Cardiovascular mortality | Stage 3 | B vs A | 0.75 (0.40–1.42) | 0.374 | 738 (78) | — |
| Complete-case | Cardiovascular mortality | Stage 3 | D vs C | 1.85 (0.54–6.28) | 0.324 | 738 (78) | — |
| Complete-case | Cardiovascular mortality | Stage 4 | B vs A | 1.09 (0.78–1.52) | 0.608 | 3,087 (509) | — |
| Complete-case | Cardiovascular mortality | Stage 4 | D vs C | 0.98 (0.70–1.37) | 0.921 | 3,087 (509) | — |
| HSI ≥36 steatosis proxy | All-cause mortality | Stage 0 | Model not estimable | — | — | — | Sparse cell: Observed C n=1/events=0; D n=0/events=0 |
| HSI ≥36 steatosis proxy | All-cause mortality | Stage 1 | B vs A | 0.80 (0.41–1.55) | 0.503 | 2,806 (80) | — |
| HSI ≥36 steatosis proxy | All-cause mortality | Stage 1 | D vs C | — | — | 2,806 (80) | Sparse cell: Observed C n=4/events=2; D n=11/events=4 |
| HSI ≥36 steatosis proxy | All-cause mortality | Stage 2 | B vs A | 0.92 (0.83–1.02) | 0.097 | 22,506 (2,535) | — |
| HSI ≥36 steatosis proxy | All-cause mortality | Stage 2 | D vs C | 0.89 (0.70–1.13) | 0.345 | 22,506 (2,535) | — |
| HSI ≥36 steatosis proxy | All-cause mortality | Stage 3 | B vs A | 0.85 (0.62–1.17) | 0.325 | 812 (303) | — |
| HSI ≥36 steatosis proxy | All-cause mortality | Stage 3 | D vs C | 0.83 (0.46–1.53) | 0.558 | 812 (303) | — |
| HSI ≥36 steatosis proxy | All-cause mortality | Stage 4 | B vs A | 0.92 (0.77–1.09) | 0.336 | 3,346 (1,306) | — |
| HSI ≥36 steatosis proxy | All-cause mortality | Stage 4 | D vs C | 0.91 (0.71–1.17) | 0.457 | 3,346 (1,306) | — |
| FIB-4 ≥1.3 fibrosis proxy | All-cause mortality | Stage 0 | Model not estimable | — | — | — | Sparse cell: Observed C n=211/events=27; D n=0/events=0 |
| FIB-4 ≥1.3 fibrosis proxy | All-cause mortality | Stage 1 | B vs A | 1.18 (0.47–2.94) | 0.719 | 2,806 (80) | — |
| FIB-4 ≥1.3 fibrosis proxy | All-cause mortality | Stage 1 | D vs C | 1.00 (0.44–2.27) | 0.996 | 2,806 (80) | — |
| FIB-4 ≥1.3 fibrosis proxy | All-cause mortality | Stage 2 | B vs A | 1.05 (0.90–1.24) | 0.529 | 22,562 (2,541) | — |
| FIB-4 ≥1.3 fibrosis proxy | All-cause mortality | Stage 2 | D vs C | 1.03 (0.91–1.16) | 0.688 | 22,562 (2,541) | — |
| FIB-4 ≥1.3 fibrosis proxy | All-cause mortality | Stage 3 | B vs A | 0.97 (0.57–1.66) | 0.910 | 820 (304) | — |
| FIB-4 ≥1.3 fibrosis proxy | All-cause mortality | Stage 3 | D vs C | 1.26 (0.91–1.75) | 0.158 | 820 (304) | — |
| FIB-4 ≥1.3 fibrosis proxy | All-cause mortality | Stage 4 | B vs A | 1.28 (0.94–1.74) | 0.114 | 3,361 (1,309) | — |
| FIB-4 ≥1.3 fibrosis proxy | All-cause mortality | Stage 4 | D vs C | 1.17 (1.00–1.36) | 0.050 | 3,361 (1,309) | — |
| Fasting-triglyceride FLI | All-cause mortality | Stage 0 | Model not estimable | — | — | — | Sparse cell: Observed C n=0/events=0; D n=0/events=0 |
| Fasting-triglyceride FLI | All-cause mortality | Stage 1 | B vs A | 1.78 (0.54–5.86) | 0.346 | 1,202 (27) | — |
| Fasting-triglyceride FLI | All-cause mortality | Stage 1 | D vs C | — | — | 1,202 (27) | Sparse cell: Observed C n=3/events=2; D n=4/events=1 |
| Fasting-triglyceride FLI | All-cause mortality | Stage 2 | B vs A | 0.99 (0.81–1.20) | 0.896 | 7,968 (782) | — |
| Fasting-triglyceride FLI | All-cause mortality | Stage 2 | D vs C | 1.27 (0.77–2.10) | 0.349 | 7,968 (782) | — |
| Fasting-triglyceride FLI | All-cause mortality | Stage 3 | B vs A | 1.28 (0.64–2.56) | 0.488 | 279 (88) | — |
| Fasting-triglyceride FLI | All-cause mortality | Stage 3 | D vs C | 0.61 (0.23–1.59) | 0.312 | 279 (88) | — |
| Fasting-triglyceride FLI | All-cause mortality | Stage 4 | B vs A | 1.50 (1.09–2.06) | 0.013 | 1,230 (404) | — |
| Fasting-triglyceride FLI | All-cause mortality | Stage 4 | D vs C | 0.82 (0.53–1.26) | 0.357 | 1,230 (404) | — |

***Note.*** *The table reports complete-case, 2-year landmark, HSI, FIB-4, and correctly weighted fasting-triglyceride FLI analyses. Non-estimable cells retain their reason; results were not summarized as uniformly robust. FIB-4, fibrosis-4 index; FLI, fatty liver index; HSI, hepatic steatosis index; MI, multiple imputation.*

**Supplementary Table S14. Multiple-imputation targets and completed-data integrity**

**Panel A. Prespecified imputation targets**

| **Variable** | **N** | **Missing before** | **Missing before, %** | **Method** | **Missing after data set 1** |
| --- | --- | --- | --- | --- | --- |
| education | 31,317 | 26 | 0.08% | polyreg | 0 |
| pir | 31,317 | 2,550 | 8.14% | pmm | 0 |
| marital_status | 31,317 | 305 | 0.97% | polyreg | 0 |
| smoking | 31,317 | 20 | 0.06% | polyreg | 0 |

**Panel B. Completed-data integrity summary**

| **Audit item** | **Result** |
| --- | --- |
| Number of imputations | 20 |
| Iterations completed | 10 |
| Random seed | 8,303,850 |
| Participants | 31,317 |
| Completed-data rows | 626,340 |
| Targets | education; pir; marital_status; smoking |
| Protected field count | 47 |
| Protected values identical across all 20 data sets | Yes |
| Participant IDs match current cohort | Yes |
| Target missingness after all imputations | 0 |

***Note.*** *Education, income-to-poverty ratio, marital status, and smoking were the only imputation targets; marital status was an auxiliary variable and not part of the primary adjustment set. Exposure, phenotype, CKM stage, outcome, follow-up, weight, stratum, primary sampling unit, and other analysis-defining fields were protected. Survey-weighted KM and Aalen–Johansen analyses used protected fields once rather than being repeated across imputations.*

**Supplementary Table S15. Alcohol-aetiology category audit in the eligible-except-alcohol population**

| **Alcohol category** | **Weighted prevalence, % (95% CI)** | **N** | **All-cause/CV deaths** | **Person-years** | **Interpretation** |
| --- | --- | --- | --- | --- | --- |
| MASLD-range | 81.05% (80.42–81.69) | 31,317 | 4,284/1,417 | 291,392.5 | Eligible for primary MASLD analysis |
| MetALD | 4.57% (4.24–4.89) | 1,484 | 240/58 | 14,241.3 | Descriptive only; not a no-MASLD reference participant |
| ALD | 1.25% (1.10–1.41) | 501 | 78/28 | 4,652.0 | Descriptive only; not a no-MASLD reference participant |
| Unknown | 13.13% (12.55–13.70) | 6,158 | 1,485/456 | 57,149.2 | Descriptive only; not a no-MASLD reference participant |

***Note.*** *This independent eligible-except-alcohol audit applies every other final eligibility criterion while leaving alcohol unfiltered. It has a different denominator from the sequential flow, in which 8,006 participants with unknown intake and 2,329 in the MetALD/ALD range were excluded before later criteria. The two count sets are not additive or interchangeable. ALD, alcohol-related liver disease; MetALD, metabolic dysfunction- and alcohol-associated liver disease.*

**Supplementary Table S16. Feasibility of liver-related mortality ascertainment**

| **Outcome** | **Status** | **Required data** | **Prohibited shortcut** | **N** | **Events** |
| --- | --- | --- | --- | --- | --- |
| Liver-related mortality | Not estimable from public-use NDI file | Restricted-use detailed underlying/multiple-cause ICD-10 mortality data | UCOD_LEADING residual/other category must not be relabeled as liver death | — | — |

***Note.*** *The public-use NDI file provides broad UCOD_LEADING categories and does not expose the detailed underlying or multiple-cause ICD-10 codes required to identify liver-related mortality. The residual other-cause category was not relabelled as liver mortality. NDI, National Death Index.*

**Supplementary Table S17. Vibration-controlled transient-elastography proxy performance in NHANES 2017–2018**

| **Index test** | **Proxy reference** | **Metric** | **Estimate (95% CI)** | **N** | **Analysis** | **Limitation** |
| --- | --- | --- | --- | --- | --- | --- |
| FLI≥60 | CAP≥248 dB/m steatosis | Sensitivity | 0.717 (0.682–0.752) | 2,087 | Survey-weighted cross-sectional agreement in VCTE-eligible 2017-2018 participants | CAP/LSM are noninvasive proxies, not biopsy; mortality validation is not claimed |
| FLI≥60 | CAP≥248 dB/m steatosis | Specificity | 0.772 (0.725–0.819) | 1,343 | Survey-weighted cross-sectional agreement in VCTE-eligible 2017-2018 participants | CAP/LSM are noninvasive proxies, not biopsy; mortality validation is not claimed |
| FLI≥60 | CAP≥248 dB/m steatosis | PPV | 0.814 (0.783–0.845) | 1,771 | Survey-weighted cross-sectional agreement in VCTE-eligible 2017-2018 participants | CAP/LSM are noninvasive proxies, not biopsy; mortality validation is not claimed |
| FLI≥60 | CAP≥248 dB/m steatosis | NPV | 0.662 (0.619–0.706) | 1,659 | Survey-weighted cross-sectional agreement in VCTE-eligible 2017-2018 participants | CAP/LSM are noninvasive proxies, not biopsy; mortality validation is not claimed |
| NFS>0.676 | LSM≥8 kPa significant fibrosis | Sensitivity | 0.257 (0.200–0.314) | 346 | Survey-weighted cross-sectional agreement in VCTE-eligible 2017-2018 participants | CAP/LSM are noninvasive proxies, not biopsy; mortality validation is not claimed |
| NFS>0.676 | LSM≥8 kPa significant fibrosis | Specificity | 0.928 (0.915–0.941) | 3,084 | Survey-weighted cross-sectional agreement in VCTE-eligible 2017-2018 participants | CAP/LSM are noninvasive proxies, not biopsy; mortality validation is not claimed |
| NFS>0.676 | LSM≥8 kPa significant fibrosis | PPV | 0.257 (0.196–0.319) | 444 | Survey-weighted cross-sectional agreement in VCTE-eligible 2017-2018 participants | CAP/LSM are noninvasive proxies, not biopsy; mortality validation is not claimed |
| NFS>0.676 | LSM≥8 kPa significant fibrosis | NPV | 0.928 (0.914–0.943) | 2,986 | Survey-weighted cross-sectional agreement in VCTE-eligible 2017-2018 participants | CAP/LSM are noninvasive proxies, not biopsy; mortality validation is not claimed |
| NFS>0.676 | LSM≥12 kPa advanced fibrosis | Sensitivity | 0.360 (0.238–0.482) | 116 | Survey-weighted cross-sectional agreement in VCTE-eligible 2017-2018 participants | CAP/LSM are noninvasive proxies, not biopsy; mortality validation is not claimed |
| NFS>0.676 | LSM≥12 kPa advanced fibrosis | Specificity | 0.920 (0.905–0.936) | 3,314 | Survey-weighted cross-sectional agreement in VCTE-eligible 2017-2018 participants | CAP/LSM are noninvasive proxies, not biopsy; mortality validation is not claimed |
| NFS>0.676 | LSM≥12 kPa advanced fibrosis | PPV | 0.127 (0.077–0.176) | 444 | Survey-weighted cross-sectional agreement in VCTE-eligible 2017-2018 participants | CAP/LSM are noninvasive proxies, not biopsy; mortality validation is not claimed |
| NFS>0.676 | LSM≥12 kPa advanced fibrosis | NPV | 0.978 (0.970–0.986) | 2,986 | Survey-weighted cross-sectional agreement in VCTE-eligible 2017-2018 participants | CAP/LSM are noninvasive proxies, not biopsy; mortality validation is not claimed |
| FIB-4≥1.3 | LSM≥8 kPa significant fibrosis | Sensitivity | 0.309 (0.247–0.371) | 346 | Survey-weighted cross-sectional agreement in VCTE-eligible 2017-2018 participants | CAP/LSM are noninvasive proxies, not biopsy; mortality validation is not claimed |
| FIB-4≥1.3 | LSM≥8 kPa significant fibrosis | Specificity | 0.763 (0.725–0.801) | 3,084 | Survey-weighted cross-sectional agreement in VCTE-eligible 2017-2018 participants | CAP/LSM are noninvasive proxies, not biopsy; mortality validation is not claimed |
| FIB-4≥1.3 | LSM≥8 kPa significant fibrosis | PPV | 0.112 (0.085–0.139) | 1,005 | Survey-weighted cross-sectional agreement in VCTE-eligible 2017-2018 participants | CAP/LSM are noninvasive proxies, not biopsy; mortality validation is not claimed |
| FIB-4≥1.3 | LSM≥8 kPa significant fibrosis | NPV | 0.920 (0.901–0.938) | 2,425 | Survey-weighted cross-sectional agreement in VCTE-eligible 2017-2018 participants | CAP/LSM are noninvasive proxies, not biopsy; mortality validation is not claimed |
| FIB-4≥1.3 | LSM≥12 kPa advanced fibrosis | Sensitivity | 0.378 (0.244–0.511) | 116 | Survey-weighted cross-sectional agreement in VCTE-eligible 2017-2018 participants | CAP/LSM are noninvasive proxies, not biopsy; mortality validation is not claimed |
| FIB-4≥1.3 | LSM≥12 kPa advanced fibrosis | Specificity | 0.761 (0.726–0.797) | 3,314 | Survey-weighted cross-sectional agreement in VCTE-eligible 2017-2018 participants | CAP/LSM are noninvasive proxies, not biopsy; mortality validation is not claimed |
| FIB-4≥1.3 | LSM≥12 kPa advanced fibrosis | PPV | 0.048 (0.029–0.067) | 1,005 | Survey-weighted cross-sectional agreement in VCTE-eligible 2017-2018 participants | CAP/LSM are noninvasive proxies, not biopsy; mortality validation is not claimed |
| FIB-4≥1.3 | LSM≥12 kPa advanced fibrosis | NPV | 0.974 (0.966–0.983) | 2,425 | Survey-weighted cross-sectional agreement in VCTE-eligible 2017-2018 participants | CAP/LSM are noninvasive proxies, not biopsy; mortality validation is not claimed |
| FLI | CAP≥248 dB/m steatosis | Unweighted ROC AUC (DeLong) | 0.828 (0.814–0.842) | 3,430 | Unweighted diagnostic sensitivity analysis | DeLong AUC/CI ignores complex survey design and is not a mortality-prediction metric |
| NFS | LSM≥8 kPa significant fibrosis | Unweighted ROC AUC (DeLong) | 0.714 (0.686–0.742) | 3,430 | Unweighted diagnostic sensitivity analysis | DeLong AUC/CI ignores complex survey design and is not a mortality-prediction metric |
| NFS | LSM≥12 kPa advanced fibrosis | Unweighted ROC AUC (DeLong) | 0.772 (0.731–0.813) | 3,430 | Unweighted diagnostic sensitivity analysis | DeLong AUC/CI ignores complex survey design and is not a mortality-prediction metric |
| FIB-4 | LSM≥8 kPa significant fibrosis | Unweighted ROC AUC (DeLong) | 0.605 (0.573–0.637) | 3,430 | Unweighted diagnostic sensitivity analysis | DeLong AUC/CI ignores complex survey design and is not a mortality-prediction metric |
| FIB-4 | LSM≥12 kPa advanced fibrosis | Unweighted ROC AUC (DeLong) | 0.673 (0.622–0.725) | 3,430 | Unweighted diagnostic sensitivity analysis | DeLong AUC/CI ignores complex survey design and is not a mortality-prediction metric |
| FLI≥60 | CAP≥248 dB/m steatosis | Weighted kappa (descriptive) | — | 3,430 | Survey-weighted cross-sectional agreement | No design-based kappa interval asserted |
| NFS>0.676 | LSM≥8 kPa significant fibrosis | Weighted kappa (descriptive) | — | 3,430 | Survey-weighted cross-sectional agreement | No design-based kappa interval asserted |
| NFS>0.676 | LSM≥12 kPa advanced fibrosis | Weighted kappa (descriptive) | — | 3,430 | Survey-weighted cross-sectional agreement | No design-based kappa interval asserted |
| FIB-4≥1.3 | LSM≥8 kPa significant fibrosis | Weighted kappa (descriptive) | — | 3,430 | Survey-weighted cross-sectional agreement | No design-based kappa interval asserted |
| FIB-4≥1.3 | LSM≥12 kPa advanced fibrosis | Weighted kappa (descriptive) | — | 3,430 | Survey-weighted cross-sectional agreement | No design-based kappa interval asserted |

***Note.*** *Reliable VCTE required at least 10 valid stiffness measurements, liver-stiffness IQR/median <30%, and observed CAP and LSM. CAP and LSM are non-invasive proxy reference standards, not biopsy or histology. DeLong AUC intervals are unweighted and do not account for the complex survey design. AUC, area under the curve; CAP, controlled attenuation parameter; LSM, liver-stiffness measurement; VCTE, vibration-controlled transient elastography.*

**Supplementary Table S18. CAP-defined joint-phenotype counts and exploratory mortality sensitivity analysis**

**Panel A. CAP-defined joint-phenotype distribution**

| **Phenotype** | **N** | **All-cause/CV deaths** | **Person-years** | **Median follow-up, years** | **Weighted prevalence, % (95% CI)** |
| --- | --- | --- | --- | --- | --- |
| A | 1,253 | 9/2 | 2,478.3 | 2.00 | 40.75% (37.32–44.17) |
| B | 1,732 | 13/3 | 3,455.4 | 2.08 | 50.42% (47.62–53.22) |
| C | 110 | 6/1 | 204.4 | 1.71 | 1.73% (1.27–2.18) |
| D | 334 | 14/3 | 639.4 | 1.83 | 7.11% (5.94–8.28) |

**Panel B. Exploratory all-cause mortality analysis**

| **Outcome** | **Model** | **Contrast** | **HR (95% CI)** | **P value** | **N (events)** | **Status or reason** | **Limitation** |
| --- | --- | --- | --- | --- | --- | --- | --- |
| All-cause mortality | CAP-MASLD × NFS joint phenotype | Joint CAP-MASLD/NFS phenotype model not estimable | — | — | 3,429 (42) | Joint four-level adjusted CAP-MASLD/NFS model not estimable: 42 deaths; singular covariance across imputations. | Short 2017-2018 mortality follow-up; binary CAP-MASLD model retained |
| All-cause mortality | CAP-MASLD binary | CAP-MASLD Yes vs No | 1.39 (0.63–3.06) | 0.414 | 3,429 (42) | — | Short 2017-2018 mortality follow-up; exploratory sensitivity |

***Note.*** *CAP-defined MASLD required CAP ≥248 dB/m plus at least one cardiometabolic risk factor within the MASLD alcohol range, crossed with NFS >0.676. The four-level mortality model was non-estimable with only 42 deaths; the retained binary CAP-MASLD comparison is exploratory because the 2017–2018 subset had short follow-up.*

**Supplementary Figure S1. Temporal trends in the joint MASLD–NFS phenotypes. Survey-weighted prevalence estimates are shown across the ten NHANES cycles overall and within operational CKM stages 0–4. Lines and points denote phenotypes A (no MASLD/NFS-low), B (MASLD/NFS-low), C (no MASLD/NFS-high), and D (MASLD/NFS-high). Estimates are unadjusted descriptive proportions based on the cycle-appropriate MEC survey design.**

**Supplementary Figure S2. Adjusted all-cause mortality associations by operational CKM stage. Points and horizontal bars show survey-weighted hazard ratios and 95% confidence intervals combined across 20 covariate-only imputations. Stages 2–4 use the same fully adjusted four-level phenotype model with A as reference; stage 1 retains only the supported NFS-low A/B comparison, and stage 0 is structurally unsupported. This adjusted figure complements the unadjusted Kaplan-Meier curves in Figure 3.**

**Supplementary Figure S3. Adjusted cause-specific cardiovascular mortality associations by operational CKM stage. Points and horizontal bars show survey-weighted, multiply imputed cause-specific Cox hazard ratios and 95% confidence intervals; non-cardiovascular deaths are censored. Sparse stage 0-1 contrasts remain explicit in the source data. These estimates are not Fine–Gray subdistribution hazard ratios and complement the descriptive weighted Aalen-Johansen curves in Figure 4.**

**Supplementary Figure S4. Direct within-NFS-stratum MASLD contrasts for all-cause and cardiovascular mortality. B versus A compares MASLD status within NFS-low, and D versus C compares MASLD status within NFS-high. Points and horizontal bars show survey-weighted, multiply imputed hazard ratios and 95% confidence intervals. Below-null estimates are associations and do not establish a protective effect. Main Figure 6 addresses the separate exploratory D-versus-A subgroup question.**

**Supplementary Figure S5. Apparent incremental in-sample discrimination. Points and horizontal bars show apparent weighted Harrell C estimates and 95% confidence intervals for the base, +MASLD, +NFS, and +joint models overall and by operational CKM stage. These are exploratory in-sample estimates; no external validation, time-dependent AUC, Brier score, NRI, IDI, or decision-curve net benefit was estimated.**

**Supplementary Figure S6. Cardiometabolic risk-factor burden and mortality. Points and horizontal bars show hazard ratios and 95% confidence intervals on a logarithmic scale for all-cause and cardiovascular mortality. The figure includes the continuous effect per one additional cardiometabolic risk factor and prespecified B-versus-A and D-versus-C contrasts. Models use the multiply imputed survey analysis and prespecified adjustment covariates; non-estimable rows and their reasons remain in the source data.**

**Supplementary Figure S7. Prespecified mortality sensitivity analyses. Survey-weighted hazard ratios and 95% confidence intervals are displayed by outcome, contrast, and operational CKM stage for the complete-case, 2-year landmark, HSI≥36, FIB-4≥1.3, and fasting-triglyceride FLI analyses. The vertical reference line is HR=1. Non-estimable stage–contrast combinations are omitted from the plot but retained with explicit reasons in the source table.**

**Supplementary Figure S8. Cross-sectional agreement with vibration-controlled transient elastography proxies in the reliable NHANES 2017–2018 subset. Survey-weighted sensitivity, specificity, positive predictive value, and negative predictive value are shown for FLI≥60 against CAP≥248 dB/m, and for NFS>0.676 and FIB-4≥1.3 against LSM≥8 and LSM≥12 kPa. CAP and LSM are noninvasive proxy reference standards, not biopsy or histological diagnoses.**

**Supplementary Figure S9. VCTE diagnostic sensitivity analysis. Points and horizontal bars show unweighted receiver-operating-characteristic areas under the curve with DeLong 95% confidence intervals for continuous FLI, NFS, and FIB-4 against CAP≥248 dB/m and LSM≥8 or LSM≥12 kPa. The dashed reference line denotes AUC=0.5. DeLong intervals do not account for the complex survey design and are not mortality-prediction metrics.**
